# NMR Crystallographic Journey from Light to Heavy Atoms of Mercury(II)-DOTAM Complexes and Extraction of Related Structural Parameters

**DOI:** 10.1021/acs.inorgchem.5c03503

**Published:** 2025-10-16

**Authors:** Jakub Obuch, Jan Novotný, Jiří Czernek, Ivana Císařová, Petr Hermann, Radek Marek, David L. Bryce, Libor Kobera, Jiří Brus

**Affiliations:** † Institute of Macromolecular Chemistry, Czech Academy of Sciences, Heyrovského Náměstí 2, 162 00 Prague 6, Czechia; ‡ Department of Inorganic Chemistry, Faculty of Science, 37740 Charles University, Hlavova 2030/8, 128 40 Prague 2, Czechia; § CEITEC-Central European Institute of Technology, Masaryk University, Kamenice 753/5, 62500 Brno, Czechia; ∥ Department of Chemistry, Faculty of Science, Masaryk University, Kamenice 753/5, 62500 Brno, Czechia; ⊥ Department of Chemistry and Biomolecular Sciences, 6363 University of Ottawa, Ottawa, Ontario K1N 6N5, Canada

## Abstract

Complexes of macrocyclic ligands are routinely used as MRI contrast agents and radionuclide carriers for PET and SPECT diagnostics and radiotherapy. This study explores the structural and electronic environments of two materials containing [Hg­(dotam)]^2+^ cations, using an integrated approach combining single-crystal X-ray diffraction (SC-XRD), multinuclear solid-state magnetic resonance (ssNMR) spectroscopy (^13^C, ^15^N, ^199^Hg), and relativistic density functional theory (DFT) calculations. SC-XRD revealed distinct coordination motifs, including octa- and heptacoordinated [Hg­(dotam)]^2+^ cations. Scalar and spin–orbit relativistic DFT computations accurately reproduced ^13^C and ^15^N chemical shifts, with a root-mean-square deviation of ∼0.7 ppm for ^13^C and ∼4.8 ppm for ^15^N, highlighting the importance of relativistic heavy atom effects. For ^199^Hg NMR, relativistic cluster-based methods (ADF/ReSpect) outperformed nonrelativistic approaches. An empirical regression model (*χ̅*) linked ^199^Hg shifts to the coordination number (*CN*) and averaged donor electronegativity (χ̅) (*R* ^2^ = 0.86), enabling rapid structural inference. The isotropic ^199^Hg shift correlates with the charge on the Hg atom, influencing the *p*-type frontier molecular orbitals and their paramagnetic contributions to NMR shielding. This work highlights the potential of ^199^Hg NMR as a structural descriptor and offers a strategy for NMR crystallography involving heavy elements with possible implications for catalysis, ionic liquids, and Hg-based pharmaceuticals.

## Graphical abstract

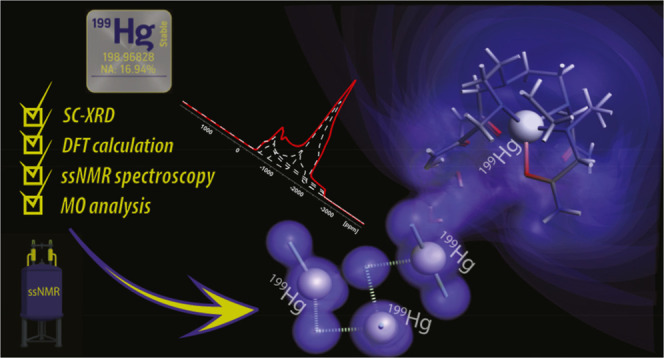

## Introduction

Inorganic–organic hybrid materials consist of two parts, inorganic and organic, intermingled on a molecular scale. These two parts are typically connected via weak interactions, such as hydrogen bonds or van der Waals forces.^1^ Such materials combine the intrinsic properties of both constituents into a single material. Moreover, these materials may even exhibit synergistic effects, which makes them a prospective choice for the preparation of new advanced materials.^2^ These compounds are studied in numerous fields, e.g., as potential advanced optical materials,^3^ semiconductors,^4^ energy materials,^5,6^ or catalysts.^7–10^

Macrocyclic ligands derived from large cyclic polyamines, often decorated on the amine groups by coordinating pendant arms, strongly coordinate a wide variety of metal ions.^11^ These complexes are widely used in medicine and biology as, e.g., MRI contrast agents (mostly Gd­(III)),^11,12^ NMR/MRI shift probes (paramagnetic metal ions),^13–15^ fluorescence probes (Ln­(III) ions),^16^ or probes for imaging and therapy (various metal radioisotopes).^17,18^ Surprisingly, Hg­(II) complexes of macrocyclic ligands have been investigated less frequently. While the majority of studies on Hg­(II) complexes of macrocyclic ligands have focused on the formation of the complex to remove or detect toxic Hg­(II) ions in the environment,^19,20^ or in vivo,^21^ the stability and inertness of these complexes also permit the safe employment of toxic heavy metal ions in vivo. A dimercury­(II) complex of ditopic *p*-xylyl-bis­(cyclen) demonstrated a pronounced affinity for thymidine triphosphate, which could be employed to sense its concentrations on a low-micrometer scale, potentially useful for monitoring cell division.^22^ Recently, Hg radioisotopes ^197*m*/197g^Hg have become emerging isotopes for potential theranostic applications, γ radiation for SPECT (SPECT = single-photon emission computed tomography) diagnostics, and Auger electrons for radiotherapy.^23^ Although the parent ligands H_4_dota and its tetraamide dotam (H_4_dota = 2,2′,2″,2‴-(1,4,7,10-tetraazacyclododecane-1,4,7,10-tetrayl)­tetraacetic acid, dotam = 2,2′,2″,2‴-(1,4,7,10-tetraazacyclododecane-1,4,7,10-tetrayl)­tetraacetamide) were shown to be ineffective for complexation of ^197m/197g^Hg­(II) under radiochemical conditions, several of their derivatives showed an increased radiochemical incorporation.^24,25^ However, detailed structural studies of Hg­(II) coordination chemistry with these macrocyclic ligands are rare.^25,26^

On the other hand, a “classical” coordination chemistry of Hg­(II) has dealt with chloromercurate­(II) (poly)­anions. Compounds with such anions have demonstrated various interesting properties stemming from their diverse molecular structures (chloride anion can coordinate up to three different Hg­(II), thus allowing formation of more complex clusters).^27–29^ Bis­(diethylammonium) tetrachloromercurate­(II) was studied for its potential use as a nonlinear optical material with efficiency of second-harmonics generation that is 1.5 times greater than that of the commonly used reference, potassium dihydrogen phosphate.^30^ Other compounds containing chloromercurate anions proved to be easily accessible ionic liquids.^31,32^ Multiple materials containing a binuclear [Hg_2_Cl_6_]^2–^ anion were studied as potential dielectric switches, and some of them have shown great reversibility and high contrast between on and off states.^33,34^ These findings demonstrate the wide applicability of compounds containing chloromercurate­(II) anions.

In this study, we present two inorganic–organic hybrid materials composed of a Hg­(II) complex of a macrocyclic ligand dotam and monomeric/polymeric chloromercurate anions according to the published procedure.^35^ To gain detailed insights into the structural characteristics of these compounds, we employed single-crystal X-ray diffraction (SC-XRD) and multinuclear solid-state NMR spectroscopy (ssNMR) together with DFT calculations. ^199^Hg NMR spectroscopy has been rarely used to probe the structure of related compounds due to significant chemical shift anisotropy (CSA) combined with extremely long *T*_1_ relaxation times. Moreover, DFT calculations on Hg atoms in solids present challenges that are typically not encountered in ″conventional″ quantum chemical calculations.^35^ In spite of these challenges, several studies utilized ^199^Hg ssNMR,^35–37^ and some ^199^Hg NMR studies focused on Hg halides also employed DFT calculations.^38–40^ However, to the best of our knowledge, no such study has been conducted on hybrid inorganic–organic materials containing a macrocyclic ligand combined with chloromercurate anions. Moreover, understanding the nature of the Hg­(II) ions in the investigated inorganic–organic hybrid materials, in the context of previously published ^199^Hg NMR data, opens up new possibilities to gain important structural information from experimental NMR data for unknown samples.

## Results and Discussion

### Synthesis

The Hg­(II) complex of dotam was prepared by the reaction of dotam with HgCl_2_ in aq. solution. Initially, in an attempt to prepare the chloride salt of the [Hg­(dotam)]^2+^ cation, a 1:1 ligand/HgCl_2_ ratio was used. However, upon crystallization, the (HgCl_4_)^2–^ salt of the Hg­(II) complex, compound **1**, was obtained in only <50% yield (based on dotam). Therefore, a larger excess of HgCl_2_ was used to improve the yield. Surprisingly, depending on the excess of HgCl_2_ used, different solid phases were obtained after crystallization. Two different phases, compounds **1** and **2**, were reproducibly prepared when the ratios dotam/HgCl_2_ = 1:2 and 1:4, respectively, were used. Both compounds were prepared in the form of single crystals suitable for SC-XRD and ssNMR. If an intermediate dotam/HgCl_2_ ratio was used during the synthesis, the HgCl_2_-“richer” compound **2** always crystallized first and the second crystallization from the mother liquor yielded the HgCl_2_-“poorer” compound **1**.

### Single-Crystal X-ray Diffraction (SC-XRD) Analysis

To date, nine structures of the Hg­(II) complex of cyclen derivatives are known (CSD database, version 6.00, April 2025). Out of these structures, only two contain derivatives with coordinating pendant arms.^25,26^ Our compound **1** contains two crystallographically independent [Hg­(dotam)]^2+^ cations, two (HgCl_4_)^2–^ anions, and one water molecule (Figure 1a). One [Hg­(dotam)]^2+^ cation features an *octacoordinated* Hg­(II) ion with four nitrogen and four oxygen donor atoms in distorted twisted square antiprismatic [6 + 2] fashion; this coordination mode has already been observed in the analogous perchlorate salt.^26^ In the second [Hg­(dotam)]^2+^ cation, the Hg­(II) ion is *heptacoordinated* by four nitrogen and three oxygen donor atoms, similar to the coordination mode in [Hg­(H_2_dota)], albeit the coordination polyhedron in our structure is more regular due to the coordination of three identical acetamide pendant arms.^25^ The Hg­(II) in the (HgCl_4_)^2–^ anions is *tetracoordinated* by chloride anions in a close to regular tetrahedral geometry.

**Figure 1:**
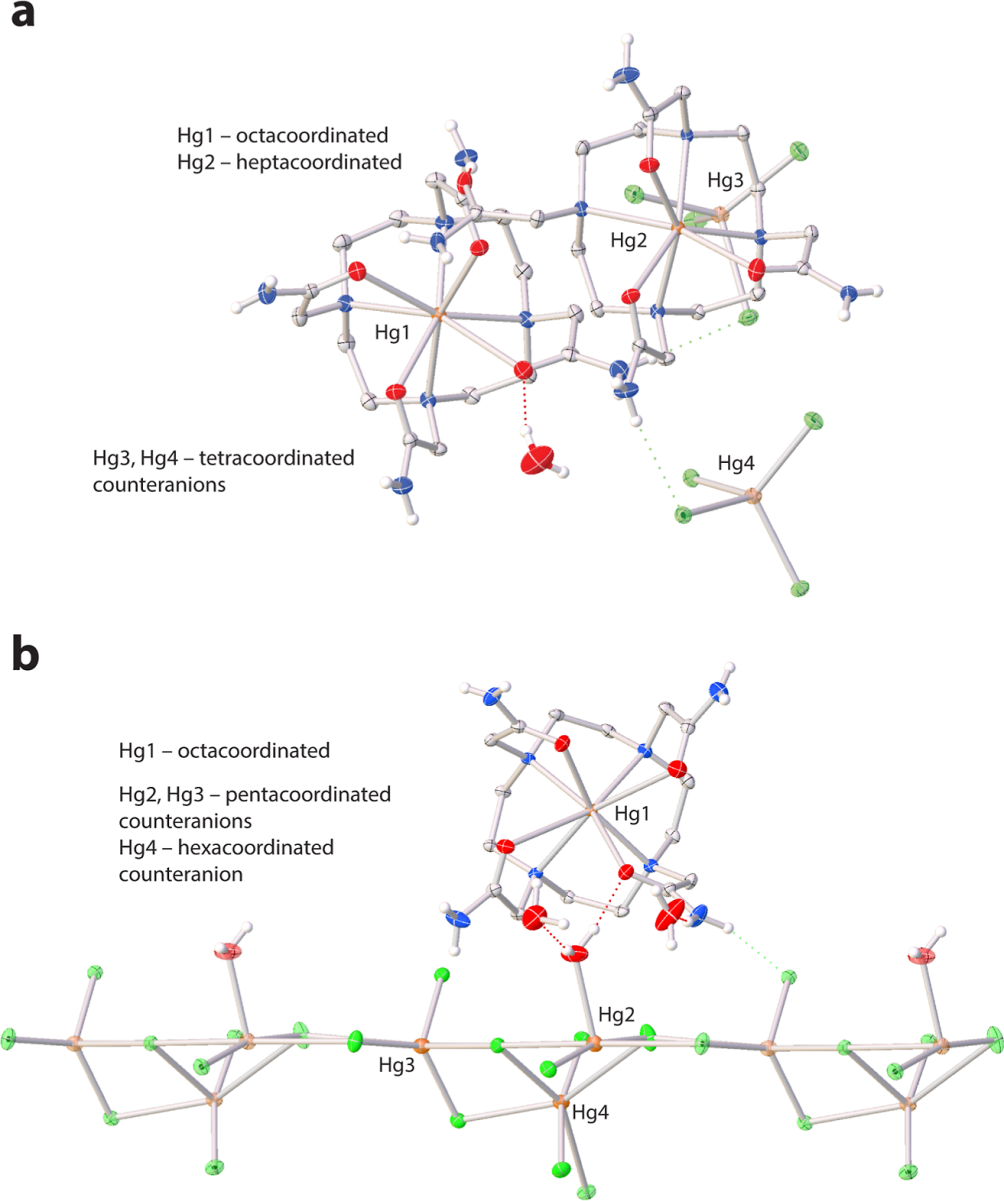
Molecular structure of the asymmetric unit in (a) compound **1** and (b) compound **2**. Carbon-bound hydrogen atoms have been omitted for the sake of clarity. Dashed lines represent hydrogen bonds. Thermal ellipsoids are drawn at the 50% probability level. Color code: gray (C), blue (N), red (O), orange (Hg), green (Cl), white (H).

The asymmetric unit of compound **2** contains the [Hg­(dotam)]^2+^ complex cation, the [Hg_3_Cl_8_(H_2_O)]^2–^ complex anion, and two water molecules (Figure 1b). The central Hg­(II) ion is *octacoordinated* by four nitrogen and four oxygen donor atoms in distorted twisted square antiprismatic [6 + 2] fashion.^26^ The Hg­(II) ions in the [Hg_3_Cl_8_(H_2_O)]^2–^ complex anion are either *pentacoordinated* and/or *hexacoordinated*.

Comparison of lengths of the coordination bonds of the [Hg­(dotam)]^2+^ cations in compounds **1** and **2** with literature data is given in Table 1. Torsion angles of the octacoordinated [Hg­(dotam)]^2+^ cations range (in absolute values) from 21.4° to 25.9°, compared to 23.5–26.5 for [Hg­(dotam)]^2+^ in ref 26. These torsion angles are typical for a twisted square antiprismatic geometry. More detailed discussion of geometrical parameters is given in SI.

**Table 1:** Comparison of the Coordination Bond Lengths (Å) in the Present Structures with the Literature Data

	Compnd. **1A** ^a^	Compnd. **1B** ^a^	Compnd. **2**	[Hg(H_2_dota)][Bibr ref25]	[Hg(dotam)](ClO_4_)_2_ ^26^
Hg–O distances^b^	2.429	2.341	2.419	2.301	2.391
	2.450	2.417	2.420	2.425	2.434
	2.635	2.491	2.790	2.926	2.735
	2.672	^c^	2.828	^c^	2.815
Hg–N distances^b^	2.396	2.384	2.384	2.387	2.414
	2.440	2.386	2.405	2.398	2.432
	2.457	2.404	2.437	2.479	2.453
	2.486	2.524	2.454	2.497	2.468

a Letters A and B denote the two complex cations present in compound **1**.

b Given in ascending order.

c The fourth pendant arm does not form a coordination bond to the Hg­(II) ion.

Clearly, the coordination geometry of the Hg­(II) ion varies even when coordinated by closely related, relatively rigid preorganized ligands, demonstrating its low stereochemical preference. This highlights the need to experimentally determine the coordination geometry of the Hg­(II) ion as it cannot be easily predicted.

### Solid-State NMR Spectroscopy and NMR Crystallography

Even when high-quality single-crystal X-ray diffraction data are available, locating light atoms, especially hydrogen, remains a challenge.^41^ NMR crystallography addresses this limitation by integrating NMR spectroscopy with DFT calculations to validate or refine atomic-level structural information.^42^ Traditionally, ^1^H and ^13^C chemical shifts have been compared to theoretical values derived from periodic DFT methods (by using, e.g., the CASTEP code) for known structures, with excellent agreement observed for relatively simple systems.^43–49^ However, the extension of this methodology to more complex hybrid structures, particularly those containing heavy atoms such as Hg­(II), requires careful consideration of relativistic effects and local electronic environments.

A renaissance of the NMR crystallography approach has come with the upswing of ultrawide-line NMR spectroscopy and parallel improvements of DFT methodology and software (e.g., CASTEP, ADF, ReSpect).^50–52^ However, NMR parameters of heavy nuclei in solids cannot be easily predicted using ″conventional″ quantum chemical calculations. Two primary challenges must be considered. First, it becomes essential to treat the systems relativistically, especially those containing atoms of elements with the high nuclear charge.^53,54^ Second, the crystal packing and intermolecular interactions in solids perturb the electronic environment of the investigated atoms.^55^ In this respect, two computational approaches have extensively been used: (i) a cluster approach where a limited number of neighboring molecules or ions are used to model environmental effects on the central molecule; (ii) periodic boundary conditions that explicitly simulate the periodicity in the crystal arrangement. However, this second approach has lacked a state-of-the-art implementation of the relativistic treatment. In this work, we combine the best of both approaches and note a new implementation that was published during the course of this work.^56^

A periodic approach was used to include intermolecular effects in the calculation of NMR shifts, and relativistic corrections were applied to NMR shifts obtained from DFT calculations of molecules or molecular clusters. Both scalar–relativistic (SR) and spin–orbit (SO) effects were incorporated. A detailed description of these approaches is provided in the following sections.

### NMR Crystallography of Light Atoms

Typically, a combination of ^**1**^ **H** and ^**13**^ **C NMR** spectroscopy with DFT calculations is the first step toward gaining relatively straightforward insights into organic and metal–organic systems.^57,58^ Moreover, this approach can be beneficial for refinement of positions of hydrogen atoms, which are often inaccurately determined from SC-XRD or powder X-ray diffraction model(s).^59^ Unfortunately in our cases, the 1D ^1^H DUMBO/MAS NMR technique provides poorly resolved spectra (Figure 2) due to the overlap of a high number of distinct ^1^H sites (66 and 38, for compound **1** and compound **2**, respectively) within an approximately 10 ppm range. Therefore, our focus shifted to other NMR active nuclei (^13^C, ^15^N, and ^199^Hg isotopes) present in the investigated systems.

**Figure 2:**
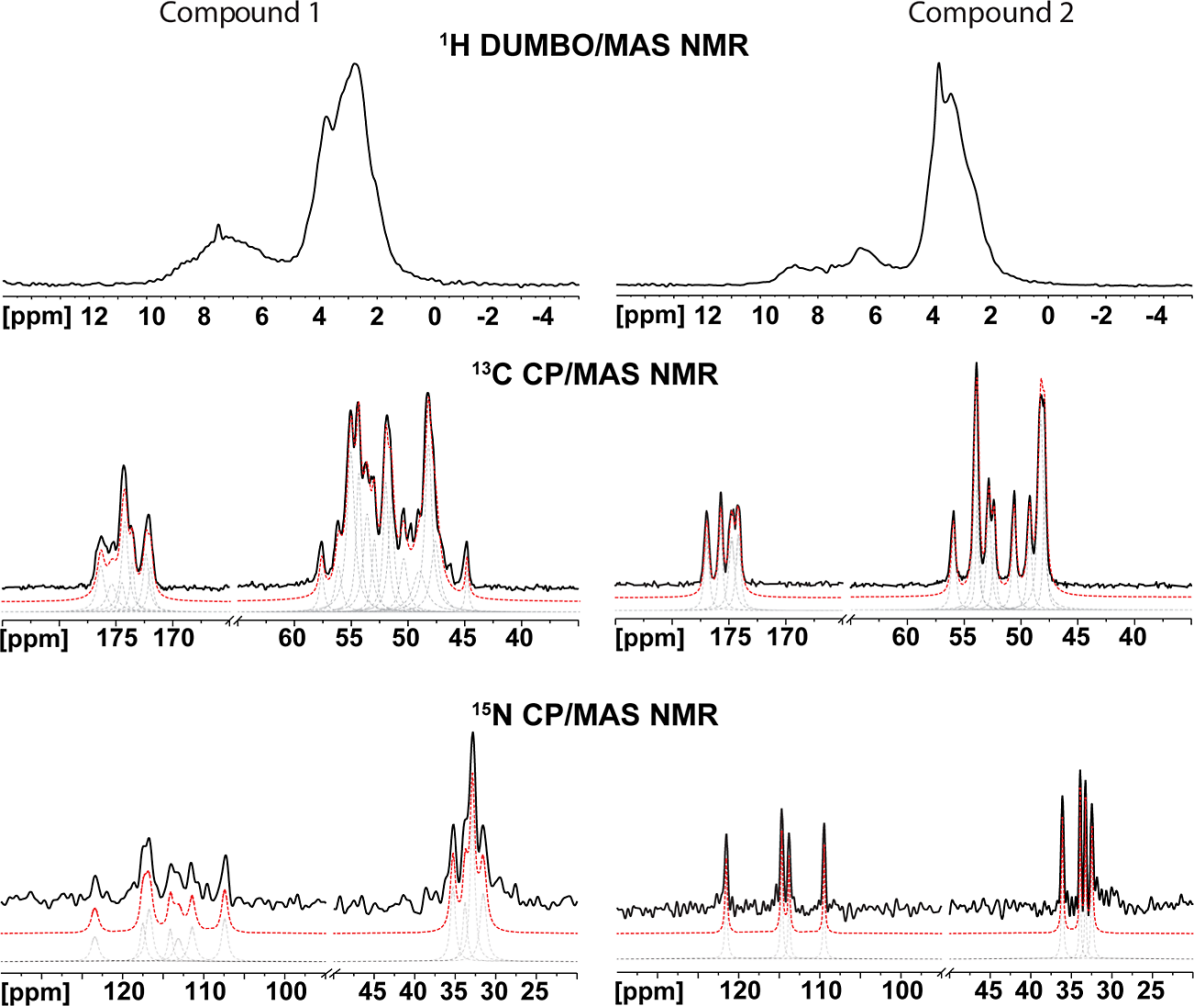
^1^H DUMBO/MAS NMR, ^13^C CP/MAS NMR, and ^15^N CP/MAS NMR spectra of compound **1** (left spectra) and compound **2** (right spectra). Experimental spectra (black solid lines), fits of individual peaks (gray dashed lines), and their sums (red dashed lines). The ^1^H DUMBO/MAS NMR spectra were recorded on a 700 MHz spectrometer, whereas ^13^C CP/MAS NMR and ^15^N CP/MAS NMR spectra were recorded on a 500 MHz spectrometer. Complete fitted parameters are given in Tables S9–S12. Figure S9 shows a comparison between the experimental and calculated ^13^C and ^15^N isotropic chemical shifts, together with the signal assignment based on Castep/SR and Castep/SR^R^/SO^R^, respectively.

Compound **2** was chosen as the initial system due to the presence of only one organic molecule in the asymmetric unit (Figure 1b), resulting in simpler NMR spectra. Because no assignment could be done experimentally, signal assignments for the ^13^C CP/MAS NMR and ^15^N CP/MAS NMR spectra were accomplished based on DFT calculations. Therefore, the experimental shifts and calculated shieldings were ordered to provide the best possible match (note that the resonance positions can be interchanged depending on the calculation method) for each computational method. Based on the root-mean-square deviation (RMSD), which was used to evaluate the experimental–theoretical match,^60^ the **Castep/SR** approach (periodic calculation including scalar-relativistic effects) provided the best results for ^13^C NMR signal assignments, with an RMSD of 0.57 ppm compared to other DFT approaches that use molecular or cluster models (Table 2). For compound **1**, which contains two organic molecules in the asymmetric unit (Figure 1a), the same approach yielded an RMSD of 0.67 ppm.

**Table 2:** ^13^C and ^15^N RMSDs Obtained by Comparison of Calculated and Experimental NMR Shifts^a^

		Castep						ReSpect	ADF
Comp.	Nucleus	NR	SR	NR/SO^R^	SR/SO^R^	NR/SR^R^	NR/SR^R^/SO^R^	SR^R^/SO^R^	SR^A^/SO^A^
**1**	^13^C	0.72	**0.67**	—	—	—	—	4.80	1.30
	^15^N	9.84	7.12	5.02	12.71	8.53	**3.80**	10.66	8.45
**2**	^13^C	1.08	**0.57**	—	—	—	—	2.52	0.75
	^15^N	11.28	8.58	6.03	8.71	10.01	4.75	4.50	8.74
overall^b^	^13^C	0.85	**0.64**	—	—	—	—	4.18	1.14
	^15^N	10.34	7.64	5.38	11.53	9.05	**4.14**	9.08	8.55

a Superscripts R and A denote application of scalar–relativistic (SR) and spin–orbit (SO) corrections calculated by ReSpect and ADF, respectively. The values without the superscript were calculated by Castep. Detailed information on computational methods is given in the Experimental Section. The best RMSD for each nucleus is set in boldface.

b These RMSDs were calculated based on data from both compounds together.

In contrast, this DFT approach was insufficient in the case of ^15^N atoms, where an RMSD of 8.58 ppm was observed for compound **2** at the **Castep/SR level**. This high RMSD value results from non-negligible HALA effects (the influence of a heavy atom on a light atom) caused by the directly bonded Hg­(II) ion.^61,62^ Consequently, several computational methods incorporating various levels of relativistic treatment were tested to calculate the ^15^N NMR shifts. The RMSD dropped from 11.3 ppm for the nonrelativistic approach in Castep down to 10.0 ppm when the SR corrections obtained from the ReSpect calculations were implemented. Incorporation of both the SR and SO corrections from the ReSpect calculations resulted in an even smaller RMSD of about 4.8 ppm (NR/SR^R^/SO^R^, see Table 2).

This NR/SR^R^/SO^R^ approach applied to compound **1** yielded an RMSD of 3.8 ppm, indicating a satisfactory agreement but also the importance of the SO-HALA effects for nitrogens neighboring heavy Hg­(II) atoms. It must be noted that the respective site assignment for ^13^C and ^15^N NMR spectra changes depending on the incorporation, or not, of relativistic effects, see Table S1. A summary of all DFT and experimental NMR shifts (with theoretical shielding values calibrated using internal regression for both systems according to the equation σ_DFT_ = *a* – δ_EXP_ where *a* is the NMR shift corresponding to σ_DFT_ = 0, see Figure S8) is provided in the Supporting Information (Tables S5–S8, SI Excel Table).

From the listed RMSD values for individual DFT approaches (Table 2), it is evident that for ^13^C atoms, which are influenced by subtle SO-HALA effects from Hg atoms, the periodic DFT method (Castep) with an implemented scalar–relativistic treatment (ZORA) provides good agreement between experimental and theoretical data. For ^15^N atoms, which are directly bonded to the Hg­(II) ion, the SO-HALA effect plays a much larger role. The best match between experimental and DFT calculated data was achieved using a combined approach where nonrelativistic periodic DFT (**Castep**-NR) results were combined with SR and SO contributions extracted from the **ReSpect** software (Castep-NR/SR^R^/SO^R^, see Table 2). Although the NMR crystallography of light atoms provided valuable insights, ^199^Hg NMR crystallography was employed in the next step to obtain detailed information about both compounds, with each containing four crystallographically distinct Hg atoms.

### NMR Crystallography of Heavy ^199^Hg Atoms

Briefly, the most commonly observed NMR-active Hg nucleus, ^199^Hg, presents several challenges during both measurement and analysis of the experimental data. First, the primary reference compound, neat dimethylmercury, is highly toxic and difficult to handle safely. This obstacle can be overcome by using either a less toxic secondary reference,^63,64^ or the unified NMR shift scale, where ^199^Hg NMR spectra are referenced to the ^1^H signal of TMS in CDCl_3_.^65^

Second, ^199^Hg signals occur over a broad frequency range (approximately 3000 ppm); therefore, the spectra often have to be acquired in a piecewise manner using variable-offset cumulative spectroscopy (VOCS) techniques.^66^ Additionally, significant chemical shift anisotropy (CSA) is commonly observed in solid-state NMR spectroscopy (except for nuclei in highly symmetrical environments), leading to broad resonances and incomplete CSA averaging by magic-angle spinning. Due to the significant breadth of these patterns, spectra are typically acquired using spin–echo-based experiments under static conditions to improve the signal-to-noise ratio with the broad peaks resolved into a series of narrow spikelets. Consequently, ^199^Hg BRAIN-CP/WURST-CPMG^67^ with VOCS as well as single-piece ^199^Hg WURST-CPMG NMR techniques were employed in this study.^68,69^

Third, the extremely long *T*_1_ relaxation times must be taken into account.^64^ Finally, analyzing the resulting spikelet ultrawide-line NMR spectra (Figure 3) poses additional challenges. The process of fitting the experimental spectra was simplified by the USS software, which extracts the envelope of the spikelet spectra, thereby enabling the application of standard fitting procedures.^70^ Fitting of complex NMR spectra often benefits from preliminary calculation of NMR parameters, but accurate DFT calculations for heavy atoms like ^199^Hg must consider relativistic effects.^38^ Here, relativistic two-component DFT calculations using the ZORA approximation were performed with the ADF program,^71^ while the recently developed approaches in the ReSpect program^52^ offer an alternative of relativistic four-component DFT approach to NMR calculations.^72^

**Figure 3:**
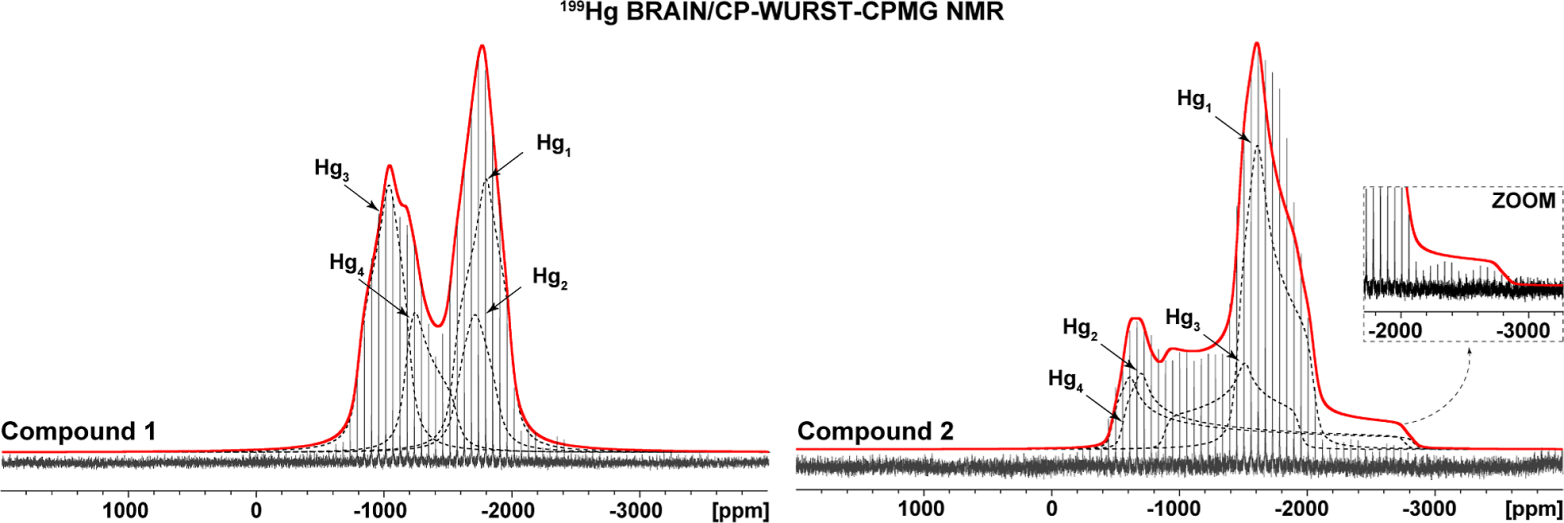
Experimental ^199^Hg ssNMR spectra (black solid line), simulations of the individual Hg sites (dashed lines), and their sum (red solid line) of compound **1** (left-hand spectrum) and compound **2** (right-hand spectrum). Both spectra were recorded on a 500 MHz spectrometer. The ^199^Hg ssNMR spectra recorded on a 700 MHz spectrometer are depicted in the Supporting Information (Figure S10).

In the investigated samples, Hg atoms can be classified into two categories: Hg­(II) coordinated by an organic ligand and Hg­(II) in inorganic chloromercurate complex anions. This structural diversity results in a broad range of ^199^Hg NMR parameters (with principal components of nuclear shielding tensors spanning approximately 2500 ppm), enabling internal calibration to convert calculated shielding components to NMR chemical shifts. To further refine NMR parameter predictions and gain deeper insight into the nature of Hg atoms, several quantum–chemical methods as implemented in CASTEP, ADF, and ReSpect were compared, and the calculated shielding values were correlated with experimental data.

In order to compare the computational methods, first, the principal components of NMR shielding tensors need to be converted into the components of the NMR shift tensors (Table 3, parameters calculated using an approach combining periodic Castep calculations with relativistic corrections analogous to the one used for light atoms are given in the SI Excel Table). This conversion was achieved using internal calibration. The calibration equations for all calculation methods were determined by linear regression (with the slope fixed to −1) on the averaged principal components of the NMR shift tensor measured on 500 and 700 MHz spectrometers of both compounds (Figure S11 and Fitting procedure). The DFT computations of the NMR parameters for the ^199^Hg nuclei are in good agreement with the experimental data; the RMSDs for isotropic shift, span, and skew are listed in Table 4. From the isotropic shift RMSDs, it is clear that a treatment with both SR and SO corrections is necessary for good prediction as the best RMSDs were obtained for Castep with SR and SO correction from ADF or ReSpect. Surprisingly, the best span estimates were obtained by nonrelativistic DFT calculations in Castep and ReSpect, perhaps suggesting a cancellation of errors. Finally, the best prediction of skew was obtained by 2c and 4c relativistic calculations.

**Table 3:** ^199^Hg NMR Parameters in the Studied Compounds Recorded on a 500 MHz Spectrometer Using the BRAIN-CP/WURST-CPMG Technique and on a 700 MHz Spectrometer Using the WURST-CPMG NMR Technique^a^

		compound **1**			compound **2**		
site		δ_iso_ (ppm)	Ω (ppm)	κ	δ_iso_ (ppm)	Ω (ppm)	κ
Hg1	Exp. (500 MHz)^b^	–1764 ± 100	401 ± 100	0.11 ± 0.1	–1699 ± 100	586 ± 200	0.46 ± 0.1
	Exp. (700 MHz)^c^	–1776 ± 100	430 ± 100	0.12 ± 0.1	–1681 ± 100	534 ± 200	0.45 ± 0.1
	Exp. (average)^d^	–1770 ± 100	416 ± 100	0.12 ± 0.1	–1690 ± 100	560 ± 200	0.46 ± 0.1
	Calc. (Castep/NR)	–1855	342	0.65	–1678	341	0.72
	Calc. (ADF)	–1791	417	0.11	–1724	477	0.63
	Calc. (ReSpect)	–1816	471	0.34	–1697	506	0.64
Hg2	Exp. (500 MHz)^b^	–1644 ± 100	407 ± 100	–0.23 ± 0.2	–1376 ± 100	2394 ± 300	0.81 ± 0.1
	Exp. (700 MHz)^c^	–1660 ± 100	396 ± 100	–0.38 ± 0.2	–1247 ± 100	2234 ± 300	0.82 ± 0.1
	Exp. (average)^d^	–1652 ± 100	402 ± 100	–0.30 ± 0.2	–1312 ± 100	2314 ± 300	0.81 ± 0.1
	Calc. (Castep/NR)	–1661	366	0.03	–1446	2201	0.87
	Calc. (ADF)	–1553	601	–0.09	–1390	3127	0.93
	Calc. (ReSpect)	–1502	789	–0.15	–1339	3151	0.89
Hg3	Exp. (500 MHz)^b^	–1013 ± 200	449 ± 100	–0.26 ± 0.1	–1412 ± 100	1153 ± 100	–0.16 ± 0.1
	Exp. (700 MHz)^c^	–1000 ± 200	435 ± 100	–0.32 ± 0.1	–1395 ± 100	1154 ± 100	–0.16 ± 0.1
	Exp. (average)^d^	–1007 ± 200	442 ± 100	–0.29 ± 0.1	–1404 ± 100	1154 ± 100	–0.16 ± 0.1
	Calc. (Castep/NR)	–1110	615	0.01	–1154	1188	–0.33
	Calc. (ADF)	–1048	528	–0.20	–1200	1398	–0.14
	Calc. (ReSpect)	–1161	640	–0.25	–1295	1406	–0.08
Hg4	Exp. (500 MHz)^b^	–1149 ± 150	515 ± 100	0.22 ± 0.4	–1322 ± 100	2486 ± 300	0.82 ± 0.1
	Exp. (700 MHz)^c^	–1262 ± 150	464 ± 100	0.5 ± 0.4	–1320 ± 100	2129 ± 300	0.82 ± 0.1
	Exp. (average)^d^	–1206 ± 150	490 ± 100	0.35 ± 0.4	–1321 ± 100	2308 ± 300	0.82 ± 0.1
	Calc. (Castep/NR)	–1017	632	0.68	–1439	2104	0.83
	Calc. (ADF)	–1000	549	0.65	–1411	3111	0.90
	Calc. (ReSpect)	–979	617	0.73	–1395	3217	0.84

a Isotropic shift: δ_iso_ = (δ_11_ + δ_22_ + δ_33_)/3; span: Ω = δ_11_ – δ_33_; skew: κ = 3­(δ_22_ – δ_iso_)/Ω.

b Experimental data obtained using the BRAIN-CP/WURST-CPMG NMR technique.

c Experimental data obtained using the WURST-CPMG NMR technique.

d Averaged experimental data obtained on 500 and 700 MHz spectrometers. The calculated princpal components of the NMR shift tensor for the ^199^Hg resonances of systems in this study were recalculated using the equations: σ_calc_ (Castep) = 5725 – δ_exp_; σ_calc_ (ADF) = 8942 – δ_exp_; σ_calc_ (ReSpect) = 11398 – δ_exp_, for parameters calculated by Castep, ADF, and ReSpect, respectively. These equations were found by linear regression with the slope parameter fixed at –1; plots of the calculated principal NMR shielding components and experimental principal NMR shift components are shown in Figure S11. The detailed information on computational methods is given in the Experimental Section.

**Table 4:** RMSDs of Experimental ^199^Hg NMR Parameters (Isotropic Shifts, Spans, Skews, and Principal Components) with Respect to NMR Parameters Calculated Using Different DFT Methods^a^

		Castep						ReSpect	ADF
Parameter	Comp.	NR	SR	NR/SO^R^	SR/SO^R^	NR/SR^R^	NR/SR^R^/SO^R^	SR^R^/SO^R^	SR^A^/SO^A^
Isotropic shift (ppm)	**1**	116	185	172	132	221	138	158	116
	**2**	154	244	76	78	259	85	67	119
	Overall^b^	136	216	133	**108**	240	**115**	**121**	**118**
Span (ppm)	**1**	119	169	162	231	199	256	229	113
	**2**	161	159	194	347	245	466	631	586
	Overall^b^	**141**	164	179	295	223	376	475	422
Skew	**1**	0.39	0.45	0.38	0.43	0.37	0.32	0.23	0.19
	**2**	0.16	0.17	0.19	0.19	0.12	0.15	0.11	0.11
	Overall^b^	0.30	0.34	0.30	0.33	0.27	0.25	**0.18**	**0.16**
Principal components (ppm)	**1**	133	205	190	173	240	179	187	127
	**2**	170	256	124	178	283	233	302	313
	Overall^b^	**152**	232	**161**	176	262	208	251	239

a Experimental parameters were obtained as an average of the values obtained at 500 and 700 MHz. Superscripts R and A denote corrections calculated by ReSpect and ADF, respectively. Corrections without the superscript were calculated by Castep. Detailed information on computational methods is given in the Experimental Section. Isotropic shift: δ_iso_ = (δ_11_ + δ_22_ + δ_33_)/3; span: Ω = δ_11_ – δ_33_; skew: κ = 3­(δ_22_ – δ_iso_)/Ω. The best RMSD for each parameter is set in boldface.

b These RMSDs were calculated based on data from both compounds together.

To briefly summarize, the appropriate computational method appears to depend on the nucleus and the parameters to be calculated. For ^13^C nuclei that are not directly bonded to the heavy Hg­(II) ion, periodic DFT calculation at the SR level is sufficient. For ^15^N nuclei directly bonded to the Hg­(II) ion, periodic DFT calculation incorporating SR and SO corrections is essential for reasonable accuracy. For accurate ^199^Hg calculations, a relativistic treatment (both SR and SO) is required for isotropic shift and skew calculations, but nonrelativistic Castep fortuitously provides the best results for span prediction. Note that the skew is the most critical parameter for fitting static UW NMR spectra with several inequivalent atoms.

Moreover, an important consideration in calculating NMR parameters of heavy atoms in molecules is the size of the cluster models used as it affects the accuracy of the predicted parameters.^40^ Calculating large clusters with a significant number of electrons is computationally expensive and often unsuccessful. On the other hand, insufficient cluster size negatively affects the accuracy of the calculations. Therefore, we suggest combining the periodic DFT calculations at the NR or SR level with the SR or SO corrections obtained using appropriate methods.

Considering the importance of accurately modeling the coordination environment for the observed NMR parameters, our attention was focused on the relationship between the NMR parameters and the structural arrangement of the Hg­(II) ion. The relationship between the NMR shift parameters and the structural arrangement provides valuable insights into the symmetry and bonding characteristics around the nucleus, offering a pathway to directly link spectral data with the molecular structure without demanding calculations.

### Relation of the ^199^Hg NMR Chemical Shift of Hg­(II) Compounds to the Coordination Environment

In this study, we aimed to evaluate the potential of ^199^Hg solid-state NMR spectroscopy, supported by relativistic DFT calculations, as a structural probe for complex hybrid systems containing Hg­(II). While single-crystal X-ray diffraction provides detailed insight into crystalline phases, many real-world Hg-containing systems of chemical or biological interest are amorphous or poorly crystalline, rendering diffraction techniques ineffective. By correlating experimentally observed ^199^Hg NMR parameters with local geometrical features obtained from crystallography and modeling, we sought to understand the sensitivity of these NMR parameters to coordination number *CN*, donor atom identity, and symmetry. Our ultimate goal is to establish a foundation for using ^199^Hg NMR as a reliable structural descriptor for amorphous or disordered Hg-containing materials, where classical structure determination tools fall short.

The isotropic chemical shift (δ_iso_) is known to be dependent on the environment of the observed nucleus, for example, the coordination number of the nucleus^73–75^ or the electronic structure conferred by the bound atoms.^76^ On the other hand, span and skew are related to the symmetry of the environment of the observed nucleus, that is, the variance of the coordination environment in the space around the nucleus. Asymmetrical coordination increases the span (Ω) of the peak, and conversely, symmetrical coordination decreases the span.^77^ Axially symmetric coordination results in an axially symmetric shielding tensor, which is manifested in the spectra as κ = ± 1. On the other hand, in the case of approximately spherically symmetric coordination, κ approaches 0.^78^ For perfect spherical symmetry, the three principal components would be equal and the skew is undefined. For a better and more complete analysis, we used ^199^Hg NMR isotropic chemical shifts of the compounds under investigation complemented with data available in the literature for various Hg­(II)-containing compounds. The compounds investigated here and the previously published compounds with their observed ^199^Hg NMR isotropic chemical shifts are summarized in Table 5.

**Table 5:** Overview of the Presently Investigated and Previously Published Compounds with Observed Isotropic ^199^Hg NMR Shifts (δ_iso_, Referenced to δ­(Me_2_Hg) = 0 ppm)

compound	δ_iso_ (ppm)	ref.
**1** ^a^	–1770, −1652, −1007, −1206	this work
**2** ^a^	–1690, −1312, −1404, −1321	this work
HgF_2_	–2826	^39,40^
HgCl_2_	–1625	^39,40^
HgBr_2_	–2394	^39,40^
HgI_2_ ^b^	–3131	^39,40^
Hg(CH_3_CO_2_)_2_	–2526	^79,80^
Hg(palmitate)_2_	–2526	^79^
Hg(CN)_2_	–1396	^40,81^
Hg(SCN)_2_	–1266	^82^
K[Hg(SCN)_3_]	–738	^82^
Hg(SeCN)_2_	–1760	^82^
(Me_4_N)_2_[Hg(S– C_6_H_4_Cl)_4_]	–485	^83,84^
Hg(S–*t*Bu)_2_	–665	^83,84^
Bu_4_N[Hg(S–Ph)_3_]	–344	^83,84^
Et_4_N[Hg(S–*t*Bu)_3_]	–157	^84^
Me_4_N[Hg_2_(S–Ph)_6_]	–551, −573	^85^
(Et_4_N)_2_[Hg(S–C_6_H_4_Ph-2)_4_]	–433	^85,86^
Ph_4_P[Hg(S–C_6_H_2_ *-i*Pr_3_-2,4,6)_3_]	–267	^85,86^
Me_4_N[Hg(S–*i*Pr)_3_]	–79	^85,87^

a Isotropic shifts are given in order from Hg1 to Hg4.

b Not used for isotropic shift analysis due to the presence of heavy atoms bound to the Hg­(II) cation.

First, the dependence of the ^199^Hg NMR isotropic chemical shift on the coordination number *CN* of the Hg­(II) ion was investigated. From Figure 4, it is seen that a systematic decrease of the ^199^Hg NMR isotropic chemical shift values from 0 to −3000 ppm clearly corresponds with the growth of the coordination number (*CN*) of Hg­(II) ions. However, there are some outlying values. Upon closer inspection of the data, the most extreme outlier is the chemical shift of HgI_2_, presumably caused by the direct coordination of the heavy iodine atoms to the Hg­(II) ion.^40^ Therefore, this compound was omitted for further analysis. Furthermore, the isotropic chemical shifts of the sulfur-containing compounds were consistently deshielded compared to the other compounds, while the isotropic chemical shift of HgF_2_ was significantly shielded compared to the other compounds.

**Figure 4:**
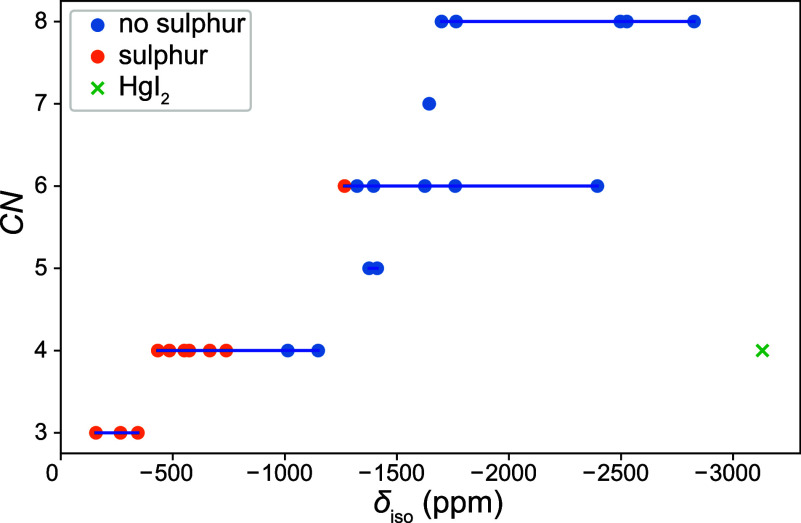
Plot of the dependence of the isotropic ^199^Hg chemical shift on the Hg­(II) coordination number (*CN*).

Second, this discrepancy led us to also consider the Pauling electronegativity (*χ*) of the donor atoms in addition to the coordination number. In the case of mixed coordination of the Hg­(II) ion, a weighted average of the electronegativities of the donor atoms was used. Therefore, a linear regression with two predictors, coordination number *CN* and average electronegativity*χ̅* of the donor atoms, was conducted, giving an empirical model expressed by eq 1 with an adjusted *R* ^2^ = 0.86 and an RMSD of the predicted isotropic shift of 298 ppm.

1 $$\delta_{pred}=-263CN-786\mathrm{\chi ̅}+2460$$

A comparison of the experimental isotropic chemical shifts and the chemical shifts predicted by linear regression is shown in Figure 5a. Most predictions are reasonably close to the experimental values, accounting for the simplicity of our model, and the residuals are approximately normally distributed, although the extremes are more pronounced. This suggests that the simple linear model with two predictors is sufficient for empirical predictions of the ^199^Hg isotropic chemical shift, provided that only light atoms coordinate the Hg­(II) ion. The histogram (Figure 5b) shows that the data set still contains some outlying values. These values were identified as chemical shifts of HgBr_2_ and Hg­(SeCN)_2_. The effect of the bromine and selenium atoms should still contribute to the ^199^Hg NMR shift. For the data set without these values, the regression results improve to an adjusted *R* ^2^ = 0.94 and RMSD = 199 ppm, see Figure S12. eq 1 indicates that the ^199^Hg isotropic chemical shift is relatively easily predicted with the knowledge of the averaged electronegativity of the donors and the number of nearby coordinated atoms.

**Figure 5:**
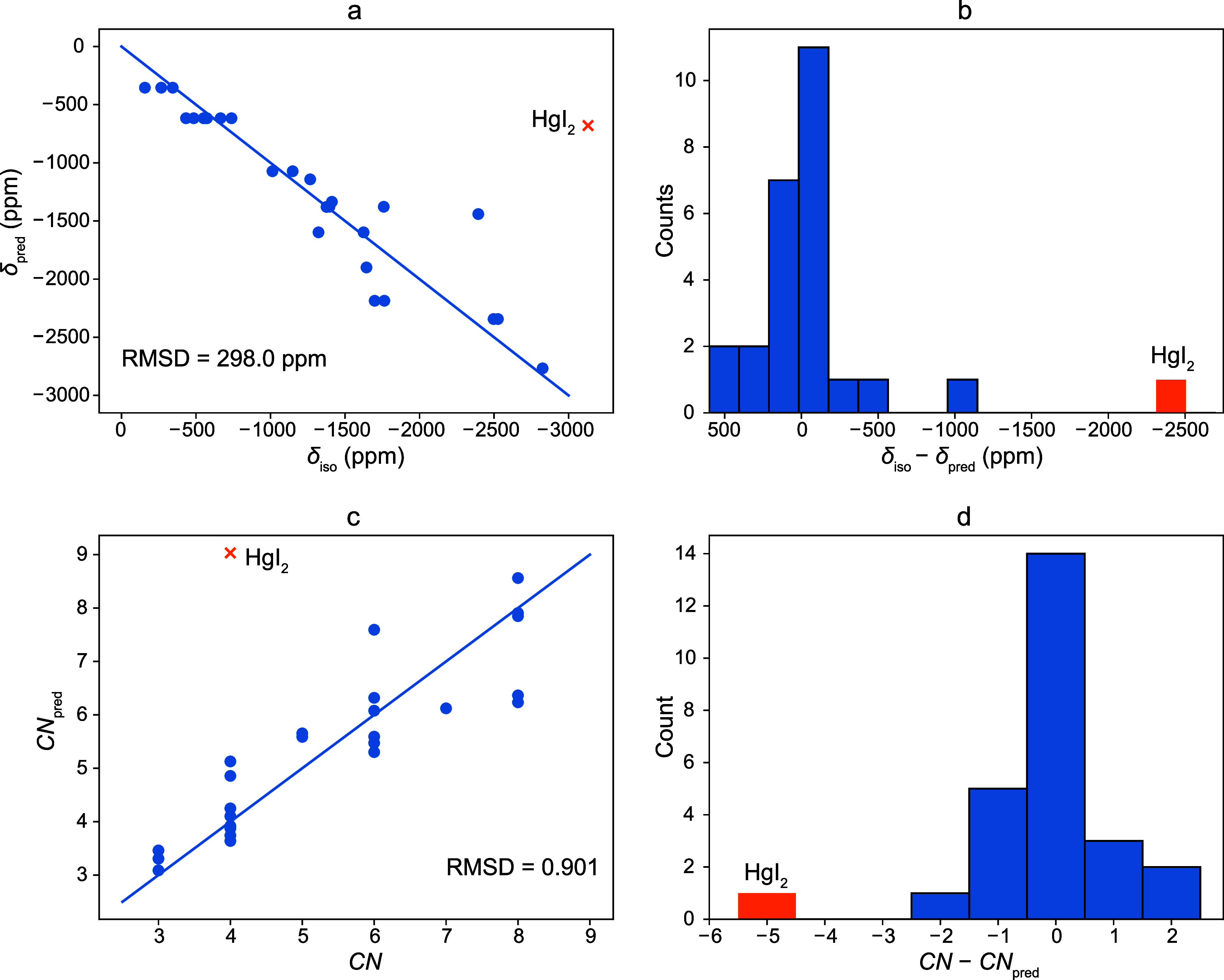
(a) Comparison of experimental isotropic ^199^Hg NMR shifts with the shifts predicted by the empirical model defined by eq 1, where the line represents an exact match. (b) Errors of prediction according to eq 1 with respect to the experimental isotropic ^199^Hg chemical shifts. (c) Comparison of the X-ray coordination number *CN* with the coordination number predicted by the empirical model given by eq 2 *CN*_pred_. (d) Errors of prediction according to eq 2 with respect to the known coordination number. The RMSDs were calculated by leave-one-out cross-validation on data sets excluding HgI_2_.

Third, a more useful, swapped model can also be derived, where the coordination number is the dependent variable, and the experimental isotropic chemical shift and the electronegativity of the donor atoms are the independent variables.

$$CN_{pred}=-0.0020\delta_{iso}-0.10\mathrm{\chi ̅}+2.50$$ 2

Briefly, in this case, the empirical model eq 2 was obtained by least-squares linear regression with an adjusted *R* ^2^ = 0.79 and an RMSD of predicted coordination numbers of 0.9. Plots of the predictions of coordination numbers are shown in Figure 5c,d. For the data set without HgI_2_, HgBr_2_, and Hg­(SeCN)_2_, the regression results improve to *R* ^2^ = 0.86 and RMSD = 0.69, see Figure S12. The potential of these models would be greatly increased if the training set was complemented with solution state data. However, the biggest issue with this is the lack of certainty in determining the coordination number in solution, most likely necessitating the use of computational methods. Although the extension of these models to solution-state systems is outside the scope of this paper, an example of such applications on literature data^25^ is given in the Supporting Information.

It is well-known that isotropic NMR shifts depend on the electronic structure and energy level gaps. To quantitatively unravel these relationships in heavy-element systems, relativistic DFT methods have become indispensable. In this manner, the (HgCl_*n*_)^2–*n*^ series was analyzed. The trend in ssNMR shielding of ^199^Hg within the (HgCl_*n*_)^2–*n*^ series (*n* = 4, 5, 6) was confirmed and analyzed using relativistic DFT calculations in ADF, see Figure 6. Counterintuitively, the Hg nucleus is more shielded with increasing coordination number, which is manifested by the more positive atomic Hirschfeld charge. A deeper investigation showed that the change of Hg shielding arises from paramagnetic contribution, and it is qualitatively reproduced already at the scalar–relativistic level (see increasing negative values of σ­(PARA) in the ZORA part of Table 6). There are two coupling mechanisms operating in the paramagnetic term of NMR shielding: transitions between occupied and vacant levels (denoted as U1) and transitions between two occupied orbitals (S1). To trace the key magnetic couplings, one needs to evaluate the contributions of the frontier MOs. A qualitative look at the energy diagram (Figure S14) revealed that mainly MOs containing nonzero coefficients of *p* AOs of the Hg­(II) ion generate deshielding effects. We can roughly conclude that with an increasing number of Cl ligands, the metal *p* contribution in occupied frontier MO space is reduced as the Cl bonding results in their energy stabilization (greater energy gap). The combination of both these parameters (smaller metal *p* character, greater energy gap) together with significantly smaller S1 deshielding contribution in (HgCl_6_)^4–^ is responsible for the observed dependence of isotropic ^199^Hg shielding. Note that this *p*-type MO contributions to NMR shielding in Hg­(II) compounds is related to the well-known fact of the closed *d*-shell stabilization in these systems and vanishing contribution of the metal *d* AO to the paramagnetic deshielding.^62^

**Figure 6:**
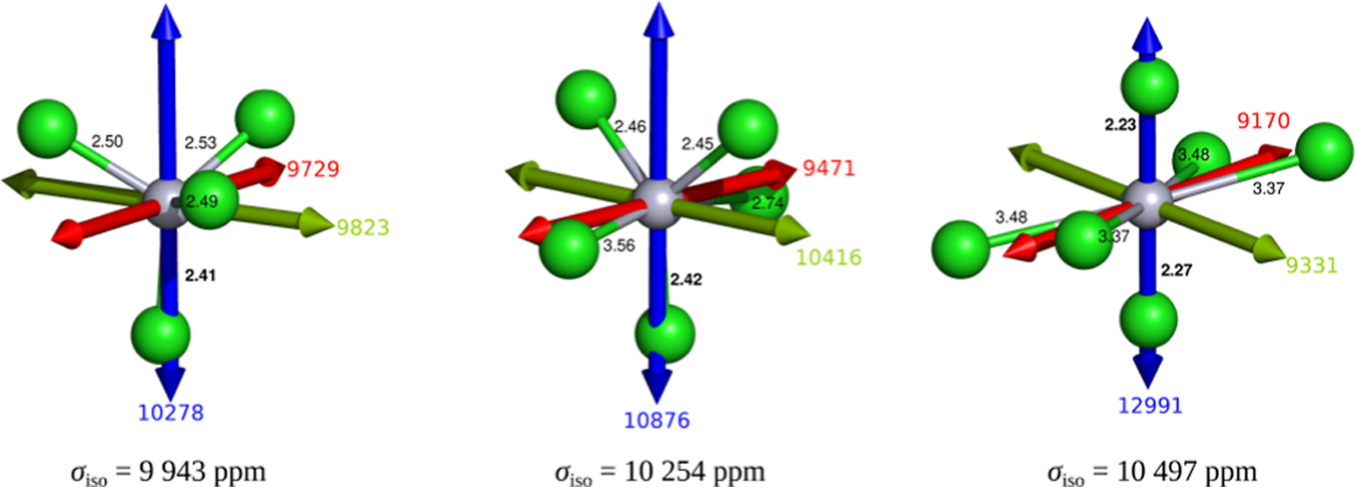
Series of (HgCl_*n*_)^2–*n*^ clusters (*n* = 4, 5, 6) and calculated principal components of ^199^Hg NMR shielding tensors. Color code for the principal components of the chemical shielding tensor is the following: blue (σ_11_), green (σ_22_), and red (σ_33_), where σ_11_ ≥ σ_22_ ≥ σ_33_. The distances between Hg and Cl atoms are denoted in black. Note that the largest components coincide with the shortest Hg–Cl bonds.

**Table 6:** Decomposition of Total Isotropic NMR Shieldings into Their Paramagnetic and Diamagnetic Contributions and Further Breakdown of the Paramagnetic Part into Transitions between Occupied and Vacant Levels (U1) and Two Occupied Levels (S1)^a^

method	cluster^b^	Hg charge (a.u.)	σ (TOT) (ppm)	σ (DIA) (ppm)	σ (PARA) (ppm)	σ (U1) (ppm)	σ (S1) (ppm)
SO-ZORA	(HgCl_4_)^2–^	0.305	9943	9952	–9	1954	–1964
	(HgCl_5_)^3–^	0.350	10254	9952	302	2565	–2262
	(HgCl_6_)^4–^	0.439	10497	9948	549	2070	–1521
ZORA	(HgCl_4_)^2–^	0.311	6619	9924	–3305	–1526	–1777
	(HgCl_5_)^3–^	0.355	6939	9924	–2985	–1131	–1852
	(HgCl_6_)^4–^	0.443	7538	9920	–2382	–1236	–1143

a The molecular orbital analysis was conducted in ADF using scalar–relativistic (ZORA) and spin–orbit (SO-ZORA) levels of theory.

b The clusters for the calculations were extracted from the structures of compound **1**: (HgCl_4_)^2–^, compound **2**: (HgCl_5_)^3–^, and HgCl_2_: (HgCl_6_)^4–^.^39^

## Conclusion

This study combines SC-XRD, ssNMR, and relativistic DFT calculations to resolve the structural and electronic properties of [Hg­(dotam)]^2+^-containing phases. Compound **1** features octacoordinated/heptacoordinated [Hg­(dotam)]^2+^ cations and isolated (HgCl_4_)^2–^ anions, while compound **2** contains an octacoordinated [Hg­(dotam)]^2+^ cation and a polymeric [Hg_3_Cl_8_(H_2_O)]^2–^ chain with mixed covalent/bridging interactions. These findings highlight stereochemical adaptability of Hg­(II) ions in forming diverse architectures.

For light nuclei (^13^C, ^15^N), scalar–relativistic periodic DFT (CASTEP) accurately predicted ^13^C shifts (RMSD <0.7 ppm), validating SC-XRD-derived carbon positions. Direct Hg­(II)–^15^N bonding necessitated spin–orbit corrections to capture heavy-atom–light-atom (HALA) effects, reducing errors from 8.6 to 4.8 ppm. ^199^Hg NMR challenges, such as extreme CSA and long *T*_1_, were addressed via BRAIN-CP/WURST-CPMG and VOCS techniques. Relativistic cluster-based DFT methods (ADF, ReSpect) outperformed nonrelativistic methods, yielding precise δ_iso_ predictions (RMSD = 67–121 ppm). The polymeric anion in compound **2** exhibited distinct ^199^Hg shifts (δ_iso_ = – 1300 to −1400 ppm) compared to the discrete (HgCl_4_)^2–^ in compound **1** (δ_iso_ = – 1000 to −1200 ppm), reflecting structural differences. An empirical linear regression model (δ_pred_ = – 263*CN* – 786*χ̅* + 2460) correlated ^199^Hg NMR shifts with coordination number (*CN*) and average donor electronegativity (*χ̅*) (*R* ^2^ = 0.86), enabling rapid structural inference in amorphous or poorly crystalline systems. This tool is critical for applications in environmental monitoring, catalysis, and biology/medicine, reducing the reliance on diffraction data. The contribution of predominantly *p*-character molecular orbitals to the paramagnetic term was identified as a main factor influencing the isotropic ^199^Hg NMR shifts. Contrary, diamagnetic term was found constant for a series of (HgCl_*n*_)^2–*n*^ clusters.

Methodologically, the hybrid computational strategy—periodic DFT for light atoms and cluster-based relativistic corrections for ^199^Hg—balances accuracy and feasibility for hybrid materials. This advances NMR crystallography as a standalone tool for heavy-metal systems. Overall, this study establishes a robust framework for heavy-metal NMR crystallography, emphasizing relativistic DFT and innovative NMR techniques. It paves the way for analyzing systems containing other heavy metal nuclei (e.g., ^207^Pb, ^205^Tl, ^119^Sn) and dynamic systems, unlocking the potential of ^199^Hg NMR spectroscopy in coordination chemistry and materials science. The sensitivity of ^199^Hg NMR to local geometry enables structural studies of amorphous or microcrystalline Hg-based materials, bridging gaps in toxicology and functional material design. The methodology’s extension to amorphous systems, where diffraction data are absent, underscores the unique role of NMR spectroscopy in heavy-metal systems analysis.

## Experimental Section

***Caution!*** *All Hg-containing compounds are toxic, and appropriate safety precautions must be taken in handling these compounds*.

### Synthesis

Ligand dotam was prepared according to the previously published procedure, and the characterization data were consistent with the previously reported literature values.^88^

#### Compound **1**

The ligand dotam (0.10 g, 0.25 mmol) was weighed into a 25 mL pear-shaped flask and dissolved in water (3 mL) and MeOH (9 mL). Then, HgCl_2_ (0.14 g; 2 equiv) was added, and the solution was stirred at 75 °C for 2 days. Afterward, the solvent was removed in vacuo. The residue was dissolved in a minimum volume of boiling water, and the solution was left to freely cool down and then kept in a refrigerator. After several days, the colorless crystalline product was collected by filtration, washed with acetone and Et_2_O, and dried in air (0.17 g; 72%). ^1^H NMR (D_2_O, pD 7.4, 400 MHz): 3.45 (8H, s, NC*H*_*2*_CONH_2_), 2.99 (8H, s, ring C*H*_*2*_), 2.80–2.63 (8H, m, ring C*H*_*2*_); ^13^C­{^1^H} (D_2_O, pD 7.4, 101 MHz): 175.1 (*C*O), 54.2 (*C*H_2_CONH_2_), 51.5–45.6 (ring *C*H_2_, broad) (Figures S5 and S6). ESI MS: (+) 601.10 (601.22, [[Hg­(dotam)]^2+^–H^+^]^+^). EA: anal. (calcd for [Hg­(dotam)]­[HgCl_4_] 0.5H_2_O): C: 19.51 (20.18), H: 3.12 (3.49), N: 10.98 (11.76), Cl: 15.64 (14.89), Hg: 44.67 (42.12).

#### Compound **2**

The ligand dotam (0.10 g, 0.25 mmol) was weighed into a 25 mL pear-shaped flask and dissolved in water (3 mL) and MeOH (9 mL). Then, HgCl_2_ (0.27 g; 4 equiv) was added, and the solution was stirred at 75 °C for 2 days. Afterward, the solvent was removed in vacuo. The residue was dissolved in minimum volume of boiling water, and the solution was left to freely cool down. After several minutes, clear long needle-like crystals formed and were collected by filtration, washed with acetone and Et_2_O, and dried in air (0.22 g; 58%). The polymeric structure of the anion is not maintained in solution; therefore, the characterization ^1^H and ^13^C­{^1^H} spectra and MS are the same as for compound **1**. ^1^H NMR (D_2_O, pD 7.4, 400 MHz): 3.45 (8H, s, NC*H*_*2*_CONH_2_), 2.99 (8H, s, ring C*H*_*2*_), 2.80–2.63 (8H, m, ring C*H*_*2*_); ^13^C­{^1^H} (D_2_O, pD 7.4, 101 MHz): 175.1 (*C*O), 54.2 (*C*H_2_CONH_2_), 51.5–45.6 (ring *C*H_2_, broad). ESI MS: (+) 601.07 (601.22, [[Hg­(dotam)]^2+^–H^+^]^+^) (Figures S5 and S7). EA: anal. (calcd for [Hg­(dotam)]­[Hg_3_Cl_8_(H_2_O)]·2H_2_O): C: 12.65 (12.47) H: 2.36 (2.49) N: 7.19 (7.27) Cl: 19.38 (18.41) Hg: 55.68 (52.09).

### Analytical Methods

Diffraction data were collected at 120 K (Cryostream Cooler, Oxford Cryosystem) on a Bruker D8 VENTURE Kappa Duo PHOTON100 diffractometer with an IμS microfocus-sealed tube using Mo-Kα (λ = 0.71073 Å) radiation. Data were analyzed using the SAINT (Bruker AXS Inc.) software package and subsequently corrected for absorption effects using the numerical method (SADABS). The structures were solved using direct methods (SHELXT2018/2)^89^ and refined with full-matrix least-squares techniques (SHELXL2019/3).^90^ All non-hydrogen atoms were refined anisotropically. All hydrogen atoms were found in the difference density map. However, hydrogen atoms bound to carbon atoms were fixed in theoretical positions using *U*_eq_(H) = 1.2 *U*_eq_(C) to keep the number of parameters low, and only hydrogen atoms bound to oxygen atoms were tried to fully refine. However, some heteroatom-bound hydrogen atoms were fixed in the original positions as the geometry during the refinement was unstable and heteroatom–hydrogen bond distances became unrealistically long or short.

Solution-state ^1^H and ^13^C­{^1^H} NMR were measured on a Bruker Avance III Neo 400 instrument (^1^H experiments at a Larmor frequency of ν­(^1^H) = 400 MHz and ^13^C experiments at a Larmor frequency of ν­(^13^C) = 101 MHz). All NMR spectra were acquired at 298 K unless stated otherwise. Spectra were referenced to the ^1^H and ^13^C signals of *t*BuOH (^1^H: 1.24 ppm and ^13^C: 30.29 ppm, respectively).

Mass spectra were recorded using a Waters ACQUITY QDa, which is part of the Waters Arc HPLC system and is shown in the Supporting Information. Data were processed using Empower 3 software. Samples were dissolved in water. As a wash solvent, 0.1% TFA in H_2_O was used.

Elemental analysis was carried out at IOCB, Prague, by combustion analysis for C–H–N and by ICP-OES for other elements.

### Solid-State NMR (ssNMR) Spectroscopy

Solid-state NMR spectra were recorded at 11.7 T using a Bruker AVANCE III HD 500 WB/US NMR spectrometer and 16.7 T using a Bruker AVANCE NEO NMR spectrometer. A 4 mm cross-polarization magic angle spinning (CP/MAS) probe was used for ^13^C and ^15^N experiments at Larmor frequencies of ν­(^13^C) = 125.78 and 50.69 MHz, respectively. The ^13^C and ^15^N spectra were collected at a spinning frequency of 10 kHz using cross-polarization periods of 2 and 7 ms, respectively. A 3.2 mm cross-polarization magic angle spinning (CP/MAS) probe was used for ^1^H DUMBO experiments at a Larmor frequency of ν­(^1^H) = 700.31 MHz and 10 kHz spinning frequency. The ^1^H, ^13^C, and ^15^N chemical shifts were calibrated using glycine (^1^H: 8.5 ppm; amide signal, ^13^C: 176.03 ppm; carbonyl signal, ^15^N: 34.35 ppm) as an external standard. A 4 mm cross-polarization magic angle spinning (CP/MAS) probe was used for ^199^Hg experiments at a Larmor frequency of ν­(^199^Hg) = 89.136 MHz, with the 3.2 mm cross-polarization magic angle spinning (CP/MAS) probe at a Larmor frequency of ν­(^199^Hg) = 125.255 MHz. The ^199^Hg BRAIN/CP WURST-CPMG NMR and WURST-CPMG NMR spectra were recorded under static conditions with a recycle delay of 16 and 600 s, respectively. The ^199^Hg BRAIN-CP/WURST-CPMG NMR experiments were carried out using a 50 μs CT-selective WURST-80 pulse at 750 kHz sweep width with 75 loops and a step of 25 kHz. The final ^199^Hg BRAIN-CP/WURST-CPMG NMR spectrum is the sum of eight subspectra. The number of scans was set to 144 for each subspectrum. The polarization transfer during the BRAIN-CP contact time was 20 ms at a 500 kHz contact pulse sweep. The ^199^Hg WURST-CPMG NMR experiments were carried out using a 50 μs CT-selective WURST-80 pulse at 1 MHz sweep width, with 75 loops as a one-piece spectrum with offset (O1) at – 175 kHz. The number of scans was 192 and 512 for the individual systems. The ^199^Hg NMR chemical shift was referenced using a unified Ξ_i_ scale as implemented in the Bruker library function “xiref”, for safety reasons. High-power ^1^H decoupling (CW) was used to eliminate heteronuclear dipolar couplings in all of the measurements. The NMR experiments were performed at a temperature of 303 K, and temperature calibration was performed to compensate for the frictional heating of the samples. All NMR spectra were processed and fitted using the TopSpin 3.5 pl7 software package.

### Computational Methods

For comparison of all computational methods with experimental values, linear regression with the slope fixed to −1 was used to prevent biased conclusions. This was done due to the slope deviating from the ideal value by as much as 0.5 for several ^15^N calculation methods, which prevented meaningful interpretation.

### Castep

The unit cell parameters of compound **1** and compound **2** were kept fixed, and all the internal coordinates were subject to optimization with respect to the crystal-lattice energy by the PW DFT (plane-waves density-functional theory) implementation in the CASTEP code.^50,91,92^ The PBE^93^ functional was applied together with the ZORA (the scalar-relativistic zeroth-order regular approximation) scheme,^94^ with the “Fine” level of settings of the CASTEP version 16.1. In particular, the PW cutoff value was 571 eV, and the Monkhorst–Pack grids^95^ used to sample the Brillouin zone were 2 × 1 × 2, no offset, 2 k-points and 3 × 2 × 2, no offset, 6 k-points for compound **1** and compound **2**, respectively. The optimized structures were then used to predict the NMR chemical shielding of all of the nuclei. The same PBE-ZORA approach as that employed in geometry optimizations was combined with the gauge-including projector augmented wave (GIPAW) method.^96,97^ The CASTEP-NMR module^50^ was used.

### ADF

All computations in the ADF suite of programs^51^ were carried out at the PBE0 level of theory. The TZ2P/all-electron (AE) basis set was used for all of the atoms in the cluster. Relativistic effects were treated with the ZORA Hamiltonian at the spin–orbit level. The quality of the Becke grid was set to good with a convergence threshold for SCF of 10^–6^. The coordinates from X-ray diffraction were used directly for the calculation of ^199^Hg magnetic shielding tensors, while proton positions were optimized prior to the calculation of ^1^H, ^13^C, and ^15^N NMR shielding tensors.^98–104^

### ReSpect

Fully relativistic (four-component) DFT calculations were performed at the mDKS/PBE0/upc-S2­(dyall-VTZ for Hg) level of theory in the ReSpect 5.2 program.^52^ The molecular models investigated here were obtained from hydrogen-optimized crystal structures. To make calculations feasible, split parts of the asymmetric unit were created and subjected to 4c-SCF (using convergence threshold 10^–6^) and subsequent GIAO NMR calculations (XALDA approximation, convergence 10^–5^).

### Regression Analysis

Analysis of the chemical shifts with respect to the structural features of the studied and literature compounds was conducted using ordinary least-squares regression as implemented in the statsmodels library in python.^105^ To prevent or detect the potential overfitting and to estimate the RMSD for data not used for training, leave-one-out cross-validation was used as implemented in the sklearn library.^106^ This cross-validation scheme was used due to the low number of data in the data set. The final models were refined using the whole data sets.

## Supplementary Material





## Data Availability

CCDC 2469569 and 2469570 contain the supplementary crystallographic data for compound **1** and **2**, respectively. The computational results are available in the ioChem-BD repository and can be accessed *via* https://doi.org/10.19061/iochem-bd-6-586. Experimental NMR data can be made available upon reasonable demand.

## Notes

All DFT calculated shieldings and calculated chemical shifts (XLSX)
X-ray crystallography, characterization data of the studied compounds, DFT calculations and NMR crystallography, ^199^Hg WURST-CPMG NMR spectra recorded on a 700 MHz spectrometer, fitting procedure of ^199^Hg NMR spectra, linear regression models omitting heavy atoms from the training set, and molecular orbital analysis diagram (PDF)
