# Supplementary material for: NMR Crystallographic Journey from Light to Heavy Atoms of Mercury(II)-DOTAM Complexes and Extraction of Related Structural Parameters
Source: Inorg Chem. 2025 Oct 16;64(42):21130–44. doi: 10.1021/acs.inorgchem.5c03503 (PMC12570143; doi:10.1021/acs.inorgchem.5c03503)
Supplement: Supplementary file 2 [file ic5c03503_si_002.pdf]

## SUPPORTING INFORMATION

# NMR Crystallographic Journey from Light to Heavy Atoms of Mercury(II)-DOTAM Complexes and Extraction of Related Structural Parameters

*Jakub Obuch*[a,b‡], *Jan Novotný*[c,d‡], *Jiří Czernek*[a], *Ivana Císařová*[b], *Petr Hermann*[b], *Radek Marek*[c,d],

*David L. Bryce*[e], *Libor Kobera*[a*], *Jiří Brus*[a]

[a]Institute of Macromolecular Chemistry, Czech Academy of Sciences, Heyrovského náměstí 2, 162 00 Prague 6, Czechia

[b]Department of Inorganic Chemistry, Faculty of Science, Charles University, Hlavova 2030/8, 128 40 Prague 2, Czechia

[c]CEITEC-Central European Institute of Technology, Masaryk University, Kamenice 753/5, 62500 Brno, Czechia

[d]Department of Chemistry, Faculty of Science, Masaryk University, Kamenice 753/5, 62500 Brno, Czechia

[e]Department of Chemistry and Biomolecular Sciences, University of Ottawa, Ottawa, Ontario, K1N 6N5, Canada

AUTHOR INFORMATION

***Corresponding Author**

Email: kobera@imc.cas.cz

Table of Contents:

X-ray Crystallography ....................................................................................................3

Characterization Data of the Studied Compounds.....................................................................13

DFT Calculations and NMR Crystallography ..........................................................................15

Fitting Procedure of $^{199}$Hg NMR Spectra..................................................................................25

Linear Regression Models Omitting Heavy Atoms from the Training Set...................................28

Application of the obtained models for solution state systems .....................................................30

Molecular Orbital Analysis Diagram..........................................................................................33

X-RAY CRYSTALLOGRAPHY

**Table S1.** Selected Experimental Data for X-ray Diffraction Studies of the Solid-state Structures of Compounds under Investigation.

| parameter | compound 1 | compound 2 |
|---|---|---|
| Formula | $2(C_{16}H_{32}HgN_8O_4)\cdot 2(Cl_4Hg)\cdot H_2O$ | $C_{16}H_{32}HgN_8O_4\cdot Cl_8H_2Hg_3O\cdot 2(H_2O)$ |
| $M_r$ | 1904.96 | 1540.50 |
| Crystal System | monoclinic | triclinic |
| Space group | $P2_1/n$ (No. 14) | $P-1$ (No. 2) |
| $a$ (Å) | 16.7931(8) | 9.5977(5) |
| $b$ (Å) | 19.9631(8) | 13.2684(7) |
| $c$ (Å) | 17.4084(8) | 14.7253(8) |
| $\alpha$ (°) | 90 | 89.396(2) |
| $\beta$ (°) | 109.843(2) | 84.730(2) |
| $\gamma$ (°) | 90 | 72.224(2) |
| $V$ (Å$^3$) | 5489.5(4) | 1777.83(16) |
| $Z$ | 4 | 2 |
| Diffrs. collected | 93881 | 144410 |
| Independent diffrs. ($R_{int}$) | 12599 (0.030) | 8158 (0.035) |
| Observed[a] diffrs. | 11978 | 8088 |
| No. of parameters | 644 | 406 |
| Observed data [$I > 2.0\ \sigma(I)$] | 11978 | 8088 |
| $N_{ref}$, $N_{par}$ | 12599, 644 | 8158, 406 |
| $R$, $wR^2$, $S$ | 0.0149, 0.0334, 1.07 | 0.0144, 0.0333, 1.13 |
| Min. and max. resd. dens. (e/Å$^3$) | −1.11, 0.87 | −1.61, 2.37 |
| CCDC ref. no. | 2469569 | 2469570 |

[a]Diffractions with $I > 2\sigma(I)$.

## Description of the Coordination Environment in $[Hg(dotam)]^{2+}$ Cations

The Hg(II) ions in the $[Hg(dotam)]^{2+}$ cations in the present compounds manifest more or less distorted twisted square antiprismatic [6+2] coordination. Whereas the octacoordinated $[Hg(dotam)]^{2+}$ cation in compound **1** is more regular than in the previously published $[Hg(dotam)]^{2+}$ structure,[1] the $[Hg(dotam)]^{2+}$ cation in compound **2** is more distorted. The nitrogen and oxygen donor atoms in the $[Hg(dotam)]^{2+}$ cations form $N_4$ (defined by the coordinated amine groups of the cyclen ring) and $O_4/O_3$ (defined by the coordinated carboxylate oxygen atoms) planes. The Hg(II) central ion lies between these two planes, almost directly on the line connecting the centroids of the two quadrilateral faces, closer to the $N_4$ plane with $Q(N_4)$ –Hg distance ($Q(N_4)$ is the centroid of the $N_4$ plane) being approximately the same for all observed cations. The $N_4$ plane is more regular in all three cations, close to square-shaped quadrilateral with all N-N-N angles deviating no more than 2° from the ideal 90° angle. Contrary, geometry of the oxygen planes is more variable. In the first $[Hg(dotam)]^{2+}$ cation of compound **1**, a rhomboidally deformed quadrilateral with two O-O-O angles close to 100° and two close to 80° was found. In the second $[Hg(dotam)]^{2+}$ cation of compound **1**, the coordinated oxygen donors form an approximately isosceles triangle. In the $[Hg(dotam)]^{2+}$ cation in compound **2**, the oxygen donors form a rhomboid, with two O-O-O angles close to 105° and two close to 75°. The coordination polyhedron around this Hg(II) central ion is therefore slightly more distorted compared to the octacoordinated Hg(II) central ion observed in compound **1**.

The heptacoordinated cation in compound **1** contains more regular $O_3$ plane compared to that in the structure of the [Hg($H_2$dota)] complex.[2] This is likely due to the coordination of three identical pendant arms instead of two different ligand types in the [Hg($H_2$dota)] complex, where the Hg(II) ion is coordinated by two deprotonated acetates and one protonated acetic acid pendant arm.

For the heptacoordinated $[Hg(dotam)]^{2+}$ cation in compound **1**, there is an alternative view of the coordination geometry of the Hg(II) ion. The donor atoms form a deformed capped octahedron, with one nitrogen donor atom and one oxygen donor atom (with angle O21B–Hg2–N4B 162.17˚) acting as the axial ligands, two nitrogen donor atoms and two oxygen donor atoms forming the square base and one nitrogen donor atom (N2B) acting as the capping ligand. The non-coordinated pendant arm is directed away from the central atom and acts as an acceptor of a hydrogen bond to an amide group of the other $[Hg(dotam)]^{2+}$ cation. However, it is more practical to consider the coordination polyhedron of Hg(II) as a twisted square antiprismatic with one oxygen donor atom removed for an easier comparison of structural variations.

Coordination polyhedron in all $[Hg(dotam)]^{2+}$ complex cations is best described as twisted square antiprism on the basis of conformations of the ring and pendant arm chelate rings. It is demonstrated by mutual twist angles of the $N_4$ and $O_4$/$O_3$ planes (**Table S3–S4**); the ideal angle is –22.5°. This arrangement is expected due to the large Hg(II) central metal ion.

**Table S2.** Selected Geometric Parameters in the Crystal Structure of Compound **1**.

| Bond | Length (Å) | Bond | Length (Å) |
|---|---|---|---|
| Hg1–O11A | 2.4287(18) | Hg2–O11B | 2.4172(17) |
| Hg1–O21A | 2.6721(18) | Hg2–O21B | 2.4910(18) |
| Hg1–O31A | 2.4496(18) | Hg2–O31B | 2.3415(18) |
| Hg1–O41A | 2.6350(18) | Hg2–N1B | 2.384(2) |
| Hg1–N1A | 2.457(2) | Hg2–N2B | 2.385(2) |
| Hg1–N2A | 2.440(2) | Hg2–N3B | 2.404(2) |
| Hg1–N3A | 2.396(2) | Hg2–N4B | 2.524(2) |
| Hg1–N4A | 2.486(2) | Hg2–$Q_N$B | 1.154 |
| Hg1–$Q_N$A | 1.199 | Hg2–$Q_O$B | 1.526 |
| Hg1–$Q_O$A | 1.390 | | |
| Hg3–Cl31 | 2.5038(6) | Hg4–Cl41 | 2.5308(6) |
| Hg3–Cl32 | 2.5579(6) | Hg4–Cl42 | 2.4955(6) |
| Hg3–Cl33 | 2.4422(7) | Hg4–Cl43 | 2.4059(7) |
| Hg3–Cl34 | 2.4500(6) | Hg4–Cl44 | 2.4892(6) |
| **Atoms** | **Angle (˚)** | **Atoms** | **Angle (˚)** |
| O41A-Hg1-O21A | 119.02(6) | O11B-Hg2-O21B | 76.38(6) |
| O31A-Hg1-O11A | 108.02(6) | O21B-Hg2-O31B | 94.47(6) |
| | | O31B-Hg2-O11B | 82.45(6) |
| O11A-O21A-O31A | 79.90(7) | O11B-O21B-O31B | 56.25(6) |
| O21A-O31A-O41A | 98.51(7) | O21B-O31B-O11B | 53.57(5) |
| O31A-O41A-O11A | 83.27(7) | O31B-O11B-O21B | 70.18(7) |
| O41A-O11A-O21A | 98.13(7) | | |
| N1A-N2A-N3A | 89.35(8) | N1B-N2B-N3B | 89.70(8) |
| N2A-N3A-N4A | 90.43(9) | N2B-N3B-N4B | 90.85(8) |
| N3A-N4A-N1A | 89.24(8) | N3B-N4B-N1B | 88.41(8) |
| N4A-N1A-N2A | 90.96(8) | N4B-N1B-N2B | 90.94(8) |
| Cl31-Hg3-Cl32 | 101.24(2) | Cl41-Hg4-Cl42 | 104.31(2) |
| Cl31-Hg3-Cl33 | 113.87(2) | Cl41-Hg4-Cl43 | 118.38(2) |
| Cl31-Hg3-Cl34 | 109.45(2) | Cl41-Hg4-Cl44 | 103.47(2) |
| Cl32-Hg3-Cl33 | 109.94(2) | Cl42-Hg4-Cl43 | 116.29(2) |
| Cl32-Hg3-Cl34 | 107.66(2) | Cl42-Hg4-Cl44 | 105.96(2) |
| Cl33-Hg3-Cl34 | 113.79(2) | Cl43-Hg4-Cl44 | 107.11(2) |
| N1A-$Q_N$A-$Q_O$A-O11A[a] | −25.65 | N1B-$Q_N$B-$Q_O$B-O11B[a] | −33.28 |
| N2A-$Q_N$A-$Q_O$A-O21A[a] | −25.43 | N2B-$Q_N$B-$Q_O$B-O21B[a] | −9.09 |
| N3A-$Q_N$A-$Q_O$A-O31A[a] | −23.60 | N3B-$Q_N$B-$Q_O$B-O31B[a] | 30.51 |
| N4A-$Q_N$A-$Q_O$A-O41A[a] | −25.94 | N4B-$Q_N$B-$Q_O$B-O41B[a] | −0.64 |

[a]$Q_N$ and $Q_O$ are centroids of the $N_4$ and $O_4/O_3$ planes.

**Table S3.** Selected Geometric Parameters in Crystal Structure of Compound **2**. Symmetry Codes: i: $x$+1, $y$, $z$, ii: $x$−1, $y$, $z$. iii: −$x$+1, −$y$, 2−$z$.

| Bond | Length (Å) | Bond | Length (Å) |
|---|---|---|---|
| Hg1–O11 | 2.828(2) | Hg2–Cl1 | 2.3049(8) |
| Hg1–O21 | 2.419(2) | Hg2–Cl2 | 2.3242(7) |
| Hg1–O31 | 2.790(2) | Hg2–Cl3 | 2.9647(7) |
| Hg1–O41 | 2.420(2) | Hg2–Cl4$^{ii}$ | 3.1972(7) |
| Hg1–N1 | 2.437(2) | Hg2–O1W | 2.450(3) |
| Hg1–N2 | 2.405(2) | Hg4–Cl7 | 2.3089(7) |
| Hg1–N3 | 2.454(2) | Hg4–Cl8 | 2.3266(7) |
| Hg1–N4 | 2.384(2) | Hg4–Cl6 | 2.9016(7) |
| Hg1–$Q_N$ | 1.161 | Hg4–Cl3 | 3.0075(7) |
| Hg1–$Q_O$ | 1.469 | Hg4–Cl8$^{iii}$ | 3.1935(8) |
| Hg3–Cl4 | 2.4166(7) | Hg4–Cl1 | 3.3497(8) |
| Hg3–Cl5 | 2.4514(7) | | |
| Hg3–Cl6 | 2.4583(7) | | |
| Hg3–Cl3 | 2.7442(7) | | |
| Hg3–Cl1$^{i}$ | 3.5545(8) | | |
| **Atoms** | **Angle (°)** | **Atoms** | **Angle (°)** |
| O11–Hg1–O31 | 120.64(6) | O21–Hg1–O41 | 100.52(7) |
| O11-O21-O31 | 104.25(8) | N1-N2-N3 | 91.1(1) |
| O21-O31-O41 | 75.75(7) | N2-N3-N4 | 88.9(1) |
| O31-O41-O11 | 106.08(8) | N3-N4-N1 | 90.1(1) |
| O41-O11-O21 | 73.31(7) | N4-N1-N2 | 89.8(1) |
| Cl4-Hg2-O1W | 93.94(8) | Cl3-Hg3-Cl4 | 91.46(2) |
| Cl1-Hg2-Cl2 | 166.15(3) | Cl3-Hg3-Cl5 | 106.13(2) |

| | | | |
|---|---|---|---|
| Cl1-Hg2-Cl3 | 95.19(2) | Cl3-Hg3-Cl6 | 93.59(2) |
| Cl6-Hg4-Cl3 | 79.951(18) | Cl4-Hg3-Cl5 | 123.72(3) |
| Cl7-Hg4-Cl8 | 165.62(3) | Cl4-Hg3-Cl6 | 130.60(3) |
| Hg2-Cl3-Hg3 | 168.29(3) | Cl5-Hg3-Cl6 | 101.76(2) |
| Hg3-Cl6-Hg4 | 92.92(2) | | |
| N1-$Q_N$-$Q_O$-O11 | −24.63 | N3-$Q_N$-$Q_O$-O31 | −23.42 |
| N2-$Q_N$-$Q_O$-O21 | −21.41 | N4-$Q_N$-$Q_O$-O41 | −23.42 |

[a]$Q_N$ and $Q_O$ are centroids of the $N_4$ and $O_4$/$O_3$ planes.

## Description of the $[Hg_3Cl_8(H_2O)]^{2-}$ Anion

The $[Hg_3Cl_8(H_2O)]^{2-}$ complex anion (**Figure S1**) has two sets of Hg–Cl distances: short covalent bonds (2.305–2.458 Å) and long crosslinking interactions (2.744–3.555 Å). This anion consists of four parts: a trigonal planar $[Hg3Cl_3]^{-}$ anion (A), a linear $Hg4Cl_2$ molecule (B), a $Cl^{-}$ anion (C), and a trigonal planar $[Hg2Cl_2(H_2O)]$ complex (D). In these formulas, subscript denotes the number of atoms, whereas regular number refers to the atom label. These parts are connected by bridging chloride ions, forming an infinite 1D chain anion with stoichiometry $([Hg_3Cl_8(H_2O)])_n^{2n-}$ along the cell axis *a*. Two chains are linked via long Hg–Cl contacts.

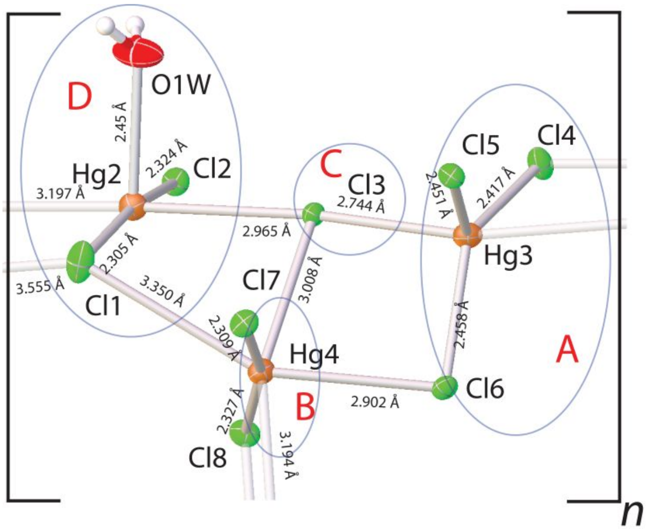


**Figure S1.** Polymeric anion in the structure of compound **2**. Color code: red (O), orange (Hg), green (Cl), white (H). Thermal ellipsoids are drawn at 50 % probability level. Circles are drawn around mercury ions to highlight ligands within a short distance of 2.5 Å.

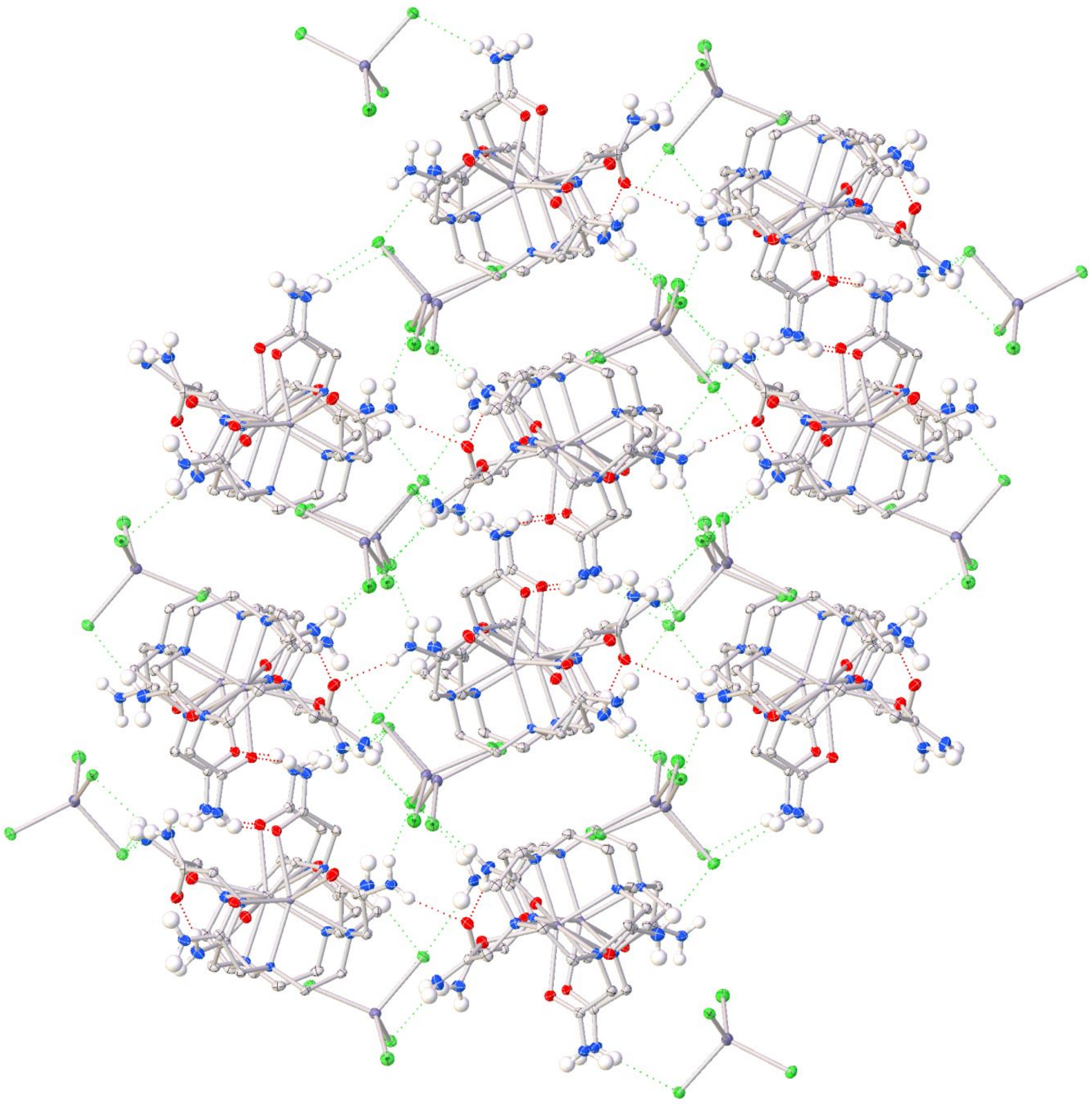

**Figure S2.** View along the crystallographic axis *a* of the crystal packing in the structure of compound **1**. For simplicity, carbon-bound hydrogen atoms and water molecules are omitted. Thermal ellipsoids are drawn at 50 % probability level. Dashed lines represent hydrogen bonds. Color code: grey (C), blue (N), red (O), violet (Hg), green (Cl), white (H).

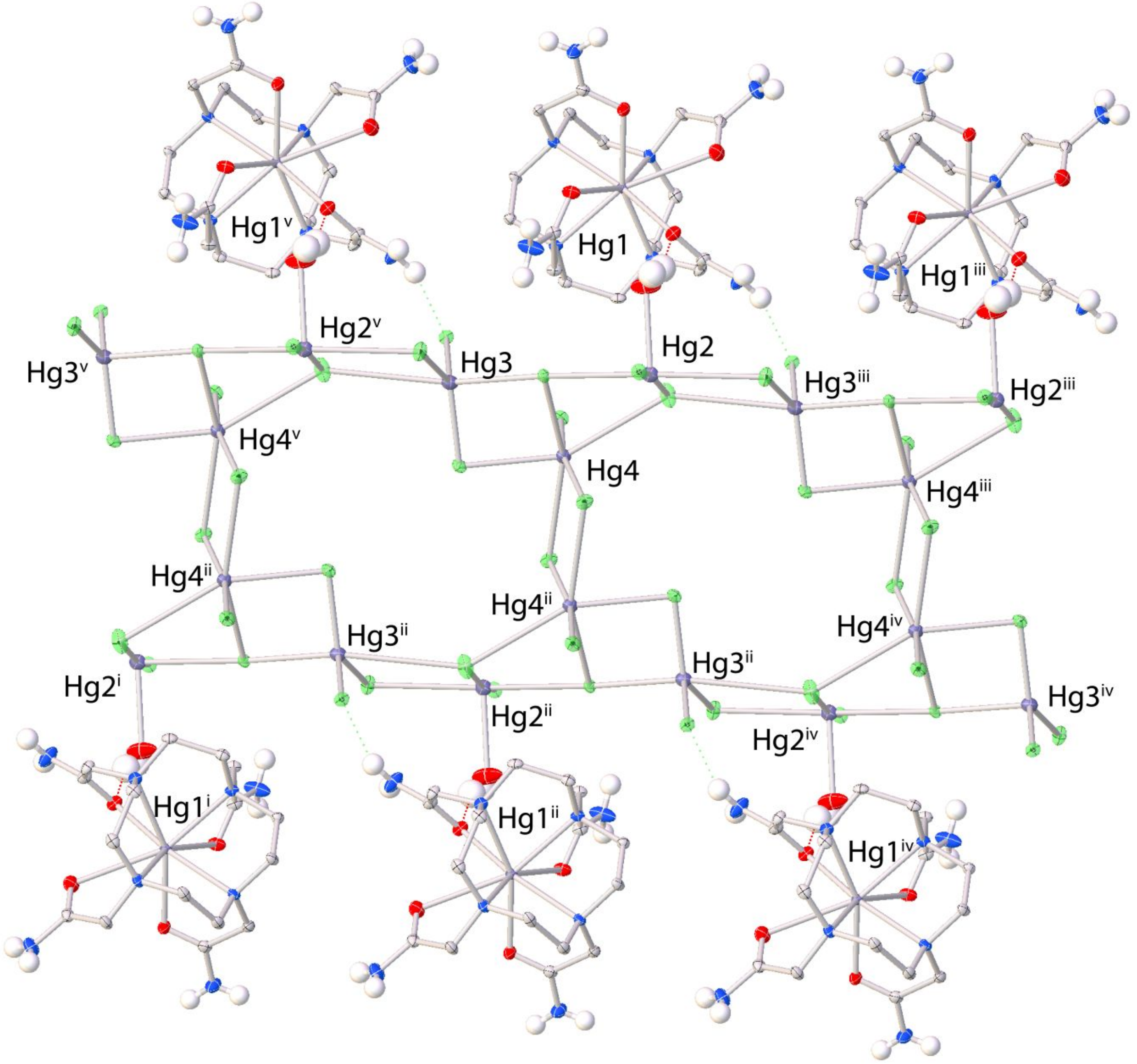


**Figure S3.** View along the crystallographic axis *c* of the crystal packing in the structure of compound **2**. For simplicity, only the mercury atom names are shown, and non-coordinated water molecules are shown only for the asymmetric unit in the original position. Carbon-bound hydrogen atoms are omitted. Thermal ellipsoids are drawn at 50 % probability level. Symmetry codes: i: $2-x$, $-y$, $2-z$, ii: $1-x$, $-y$, $2-z$, iii: $x-1$, $y$, $z$, iv: $-x$, $-y$, $2-z$, v: $x+1$, $y$, $z$. Dashed lines represent hydrogen bonds. Color code: grey (C), blue (N), red (O), violet (Hg), green (Cl), white (H).

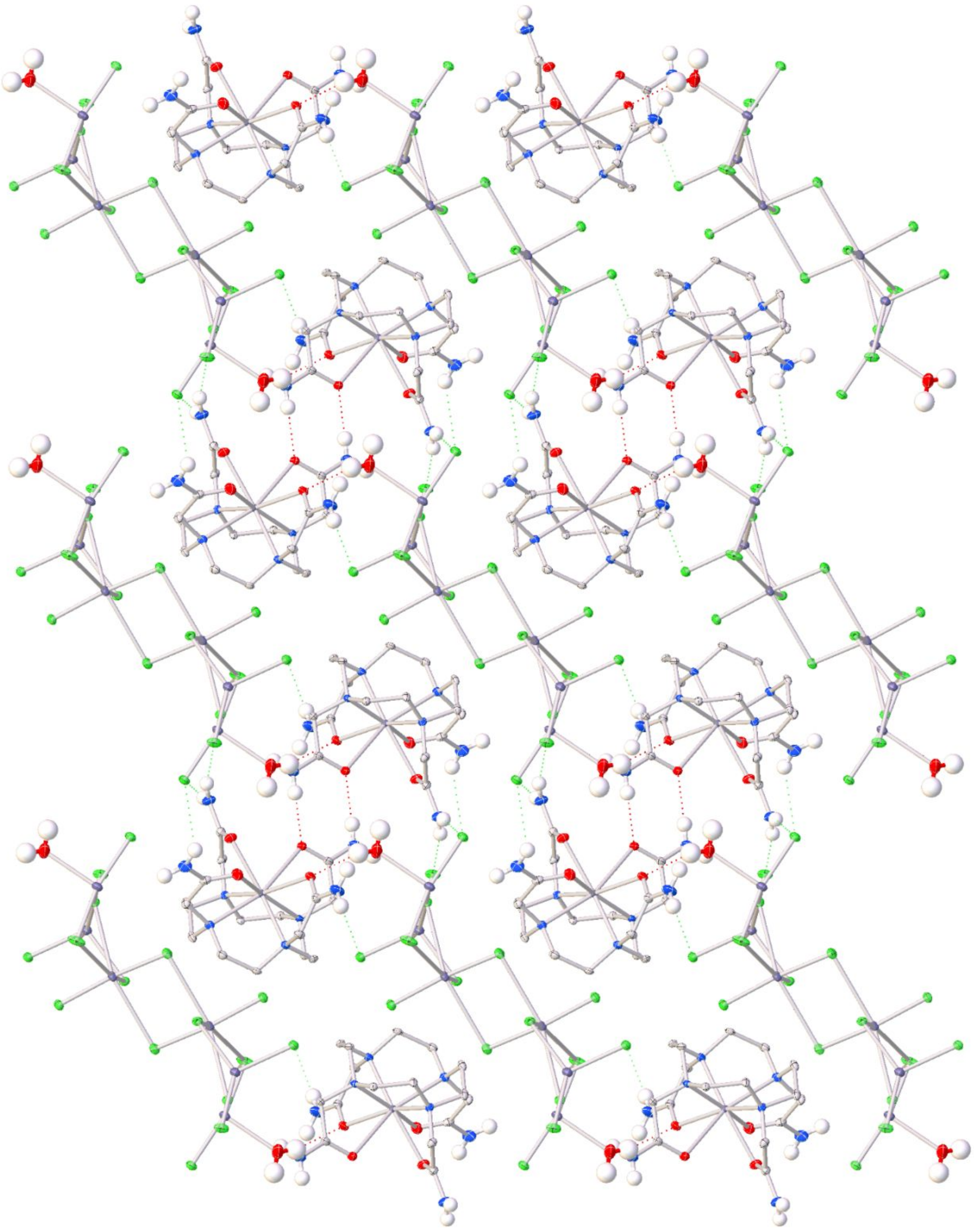

**Figure S4.** View along the crystallographic axis *a* of the crystal packing in the structure of compound **2**. For simplicity, carbon-bound hydrogen atoms are omitted. Thermal ellipsoids are drawn at 50 % probability level. The polymeric anions propagate perpendicular to the screen.

Dashed lines represent hydrogen bonds. Color code: grey (C), blue (N), red (O), violet (Hg), green (Cl), white (H).

CHARACTERIZATION DATA OF THE STUDIED COMPOUNDS

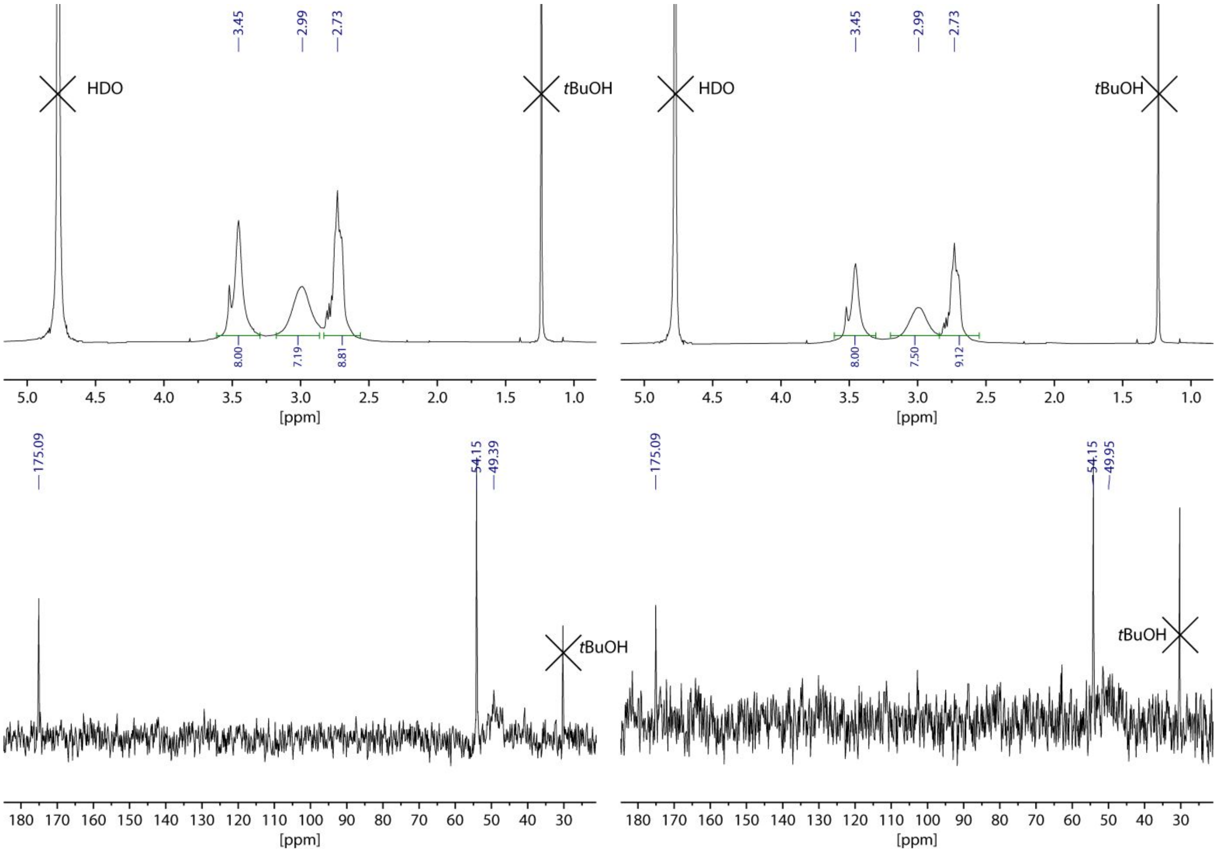


**Figure S5.** Solution NMR spectra of compound **1** (left spectra) and compound **2** (right spectra) ($D_2O$, pD 7.4) $^1H$ (400 MHz, top) and $^{13}C\{^1H\}$ (101 MHz, bottom).

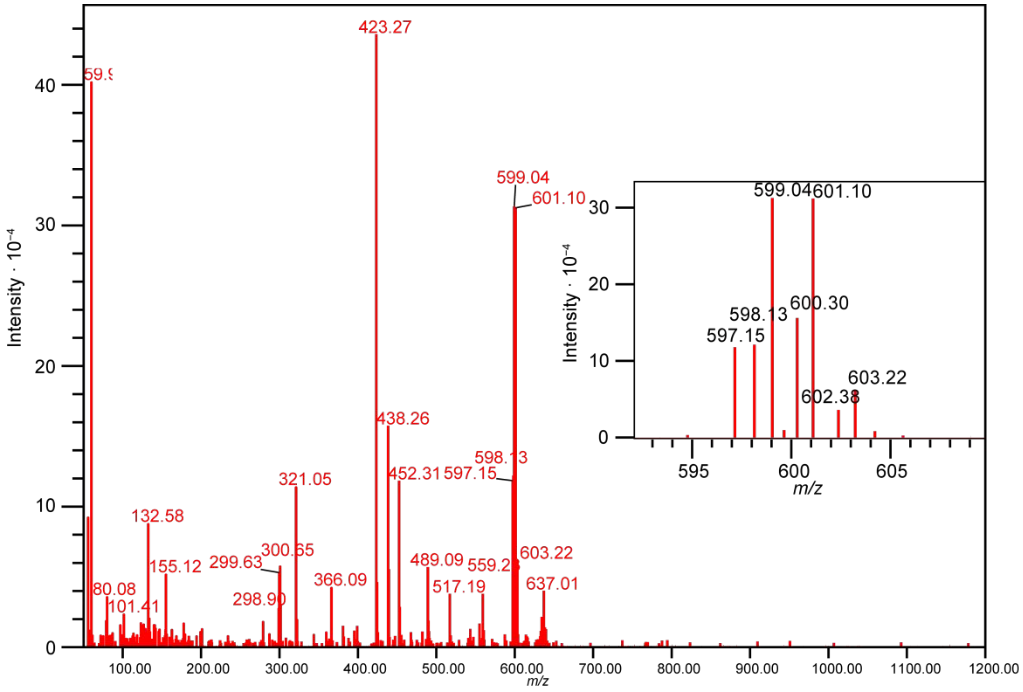


**Figure S6.** Mass spectrogram of **compound 1**. The inset shows zoom of the $[[Hg(dotam)]^{2+}-H^{+}]^{+}$ molecular ion. The peak at *m/z* = 423.27 is due to decomposition of the complex on ionization.

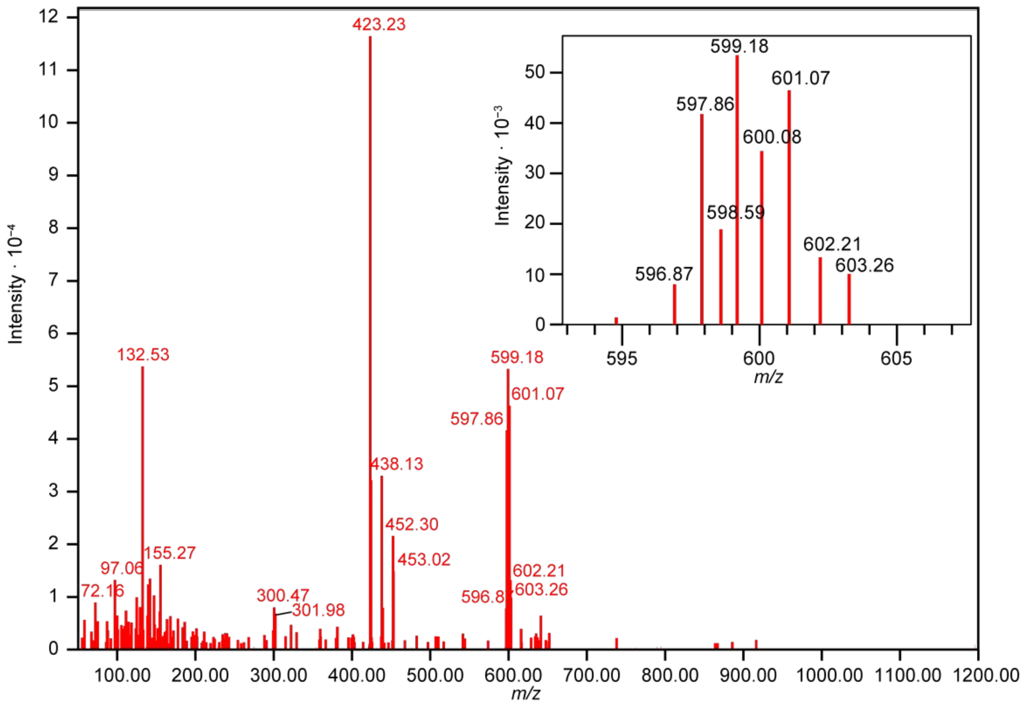


**Figure S7.** Mass spectrogram of **compound 2**. The inset shows zoom of the $[[Hg(dotam)]^{2+}-H^{+}]^{+}$ molecular ion. The peak at $m/z = 423.23$ is due to decomposition of the complex on ionization.

DFT CALCULATIONS AND NMR CRYSTALLOGRAPHY

**Table S4.** DFT-calculated $^{13}C$ Isotropic Chemical Shifts Calculated for **Compound 1**. The Site Assignment Is Done According to the Castep Results, Shifts Calculated by Other Methods are Given in Descending Order. The Calculated Shielding Constant Were Converted to Chemical Shifts Using Calibration Curves Given in **Figure S8 D–F**.

| site | $\delta_{exp}$ (ppm) | $\delta_{Castep}$ (ppm) | $\delta_{ADF}$ (ppm) | $\delta_{ReSpect}$ (ppm) |
|---|---|---|---|---|
| C12A | 176.4 | 176.4 | 178.5 | 186.5 |
| C42B | 175.4 | 175.9 | 177.9 | 183.7 |
| C32B | 174.5 | 175.3 | 176.8 | 182.6 |
| C22A | 174.2 | 174.9 | 176.4 | 179.2 |

| | | | | |
|---|---|---|---|---|
| C32A | 174.2 | 174.7 | 176.2 | 176.7 |
| C12B | 173.6 | 174.5 | 175.9 | 175.9 |
| C42A | 172.4 | 173.3 | 175.5 | 173.3 |
| C22B | 172.0 | 170.9 | 172.0 | 169.6 |
| C31B | 57.7 | 57.4 | 59.0 | 64.7 |
| C41B | 56.2 | 56.5 | 55.2 | 61.2 |
| C6B | 55.1 | 56.4 | 55.0 | 58.9 |
| C21A | 55.1 | 55.7 | 54.4 | 58.8 |
| C21B | 55.1 | 55.3 | 53.7 | 58.3 |
| C11B | 55.1 | 54.5 | 53.6 | 57.1 |
| C11A | 54.4 | 54.4 | 53.6 | 57.1 |
| C4B | 54.4 | 54.2 | 53.6 | 56.8 |
| C5B | 53.7 | 52.9 | 53.0 | 56.5 |
| C31A | 53.7 | 52.7 | 52.7 | 55.0 |
| C41A | 53.0 | 51.9 | 52.6 | 52.6 |
| C6A | 52.0 | 51.8 | 52.4 | 51.9 |
| C8B | 52.0 | 51.2 | 52.0 | 51.5 |
| C4A | 51.6 | 51.0 | 51.1 | 51.2 |
| C8A | 51.6 | 50.9 | 51.1 | 47.7 |
| C2B | 50.5 | 50.8 | 50.3 | 46.3 |
| C2A | 49.1 | 49.8 | 49.4 | 45.4 |
| C3B | 48.3 | 49.6 | 48.4 | 45.1 |
| C1A | 48.3 | 48.4 | 48.3 | 44.2 |
| C3A | 48.3 | 47.9 | 48.0 | 43.9 |
| C5A | 48.3 | 47.9 | 47.4 | 43.7 |

| | | | | |
|---|---|---|---|---|
| C7A | 47.7 | 47.7 | 47.3 | 40.4 |
| C1B | 47.7 | 47.7 | 47.0 | 38.5 |
| C7B | 44.9 | 44.5 | 43.5 | 36.9 |

**Table S5.** DFT-calculated $^{15}N$ Isotropic Chemical Shifts Calculated for Compound **1**. The Site Assignment Is Done According to the Castep Results with Scalar and Spin-orbit Corrections from ReSpect, Shifts Calculated by Other Methods Are Given in Descending Order. The Calculated Shielding Constant Were Converted to Chemical Shifts Using Calibration Curves Given in **Figure S8 G–I**.

| site | $\delta_{exp}$ (ppm) | $\delta_{Castep}$ (ppm) | $\delta_{ADF}$ (ppm) | $\delta_{ReSpect}$ (ppm) |
|---|---|---|---|---|
| N21B | 123.4 | 131.2 | 111.7 | 158.0 |
| N41B | 117.5 | 128.3 | 109.0 | 118.4 |
| N31B | 116.8 | 124.1 | 108.9 | 113.3 |
| N11B | 116.8 | 123.9 | 107.5 | 107.5 |
| N11A | 114.2 | 119.6 | 106.7 | 107.2 |
| N21A | 113.2 | 119.0 | 106.0 | 106.0 |
| N41A | 111.5 | 115.4 | 104.0 | 100.5 |
| N31A | 107.5 | 112.9 | 100.8 | 96.2 |
| N2A | 35.3 | 28.3 | 44.9 | 42.4 |
| N2B | 35.3 | 28.0 | 43.2 | 40.5 |
| N4B | 33.8 | 26.4 | 43.0 | 40.4 |
| N1B | 32.9 | 25.9 | 41.4 | 39.7 |
| N3A | 32.9 | 25.6 | 41.0 | 34.1 |

| | | | | |
|---|---|---|---|---|
| N3B | 32.9 | 25.4 | 40.9 | 33.0 |
| N1A | 31.5 | 25.1 | 40.1 | 32.7 |
| N4A | 31.5 | 23.4 | 39.1 | 32.5 |

**Table S6.** DFT-Calculated $^{13}C$ Isotropic Chemical Shifts Calculated for Compound **2**. The Site Assignment Is Done According to the Castep Results, Shifts Calculated by Other Methods Are Given in Descending Order. The Calculated Shielding Constants Were Converted to Chemical Shifts Using Calibration Curves Given in **Figure S8 D–F**.

| site | $\delta_{exp}$ (ppm) | $\delta_{Castep}$ (ppm) | $\delta_{ADF}$ (ppm) | $\delta_{ReSpect}$ (ppm) |
|---|---|---|---|---|
| C12 | 177.0 | 178.6 | 177.2 | 180.6 |
| C42 | 175.8 | 176.5 | 177.2 | 178.8 |
| C32 | 174.8 | 175.2 | 174.2 | 176.9 |
| C22 | 174.2 | 174.5 | 173.4 | 176.9 |
| C41 | 56.0 | 55.5 | 55.3 | 53.2 |
| C21 | 54.1 | 53.7 | 54.0 | 52.3 |
| C31 | 54.1 | 53.6 | 53.0 | 51.3 |
| C11 | 53.9 | 53.0 | 52.6 | 50.3 |
| C8 | 52.9 | 52.9 | 51.8 | 50.0 |
| C4 | 52.4 | 52.5 | 51.8 | 49.2 |
| C2 | 50.6 | 50.4 | 50.1 | 48.9 |
| C5 | 49.3 | 49.1 | 49.6 | 48.7 |
| C3 | 48.3 | 48.3 | 48.5 | 47.7 |
| C6 | 48.3 | 48.0 | 48.2 | 46.4 |
| C1 | 48.3 | 48.0 | 47.9 | 46.0 |

| C7 | 47.9 | 47.6 | 47.4 | 45.9 |
|---|---|---|---|---|

**Table S7.** DFT-Calculated $^{15}N$ Isotropic Chemical Shifts Calculated for Compound **2**. The Site Assignment Is Done According to the Castep Results with Scalar and Spin-orbit Corrections from ReSpect, Shifts Calculated by Other Methods are Given in Descending Order. The Calculated Shielding Constants Were Converted to Chemical Shifts Using Calibration Curves Given in **Figure S8 G–I**.

| site | $\delta_{exp}$ (ppm) | $\delta_{Castep}$ (ppm) | $\delta_{ADF}$ (ppm) | $\delta_{ReSpect}$ (ppm) |
|---|---|---|---|---|
| N41 | 121.6 | 130.8 | 107.6 | 112.3 |
| N21 | 114.7 | 123.3 | 107.2 | 112.1 |
| N11 | 113.8 | 123.0 | 106.1 | 109.2 |
| N31 | 109.5 | 118.9 | 104.5 | 103.5 |
| N1 | 36.1 | 28.6 | 45.8 | 37.9 |
| N3 | 33.9 | 25.8 | 42.2 | 36.2 |
| N2 | 33.3 | 24.9 | 40.7 | 35.5 |
| N4 | 32.5 | 24.5 | 40.1 | 32.9 |

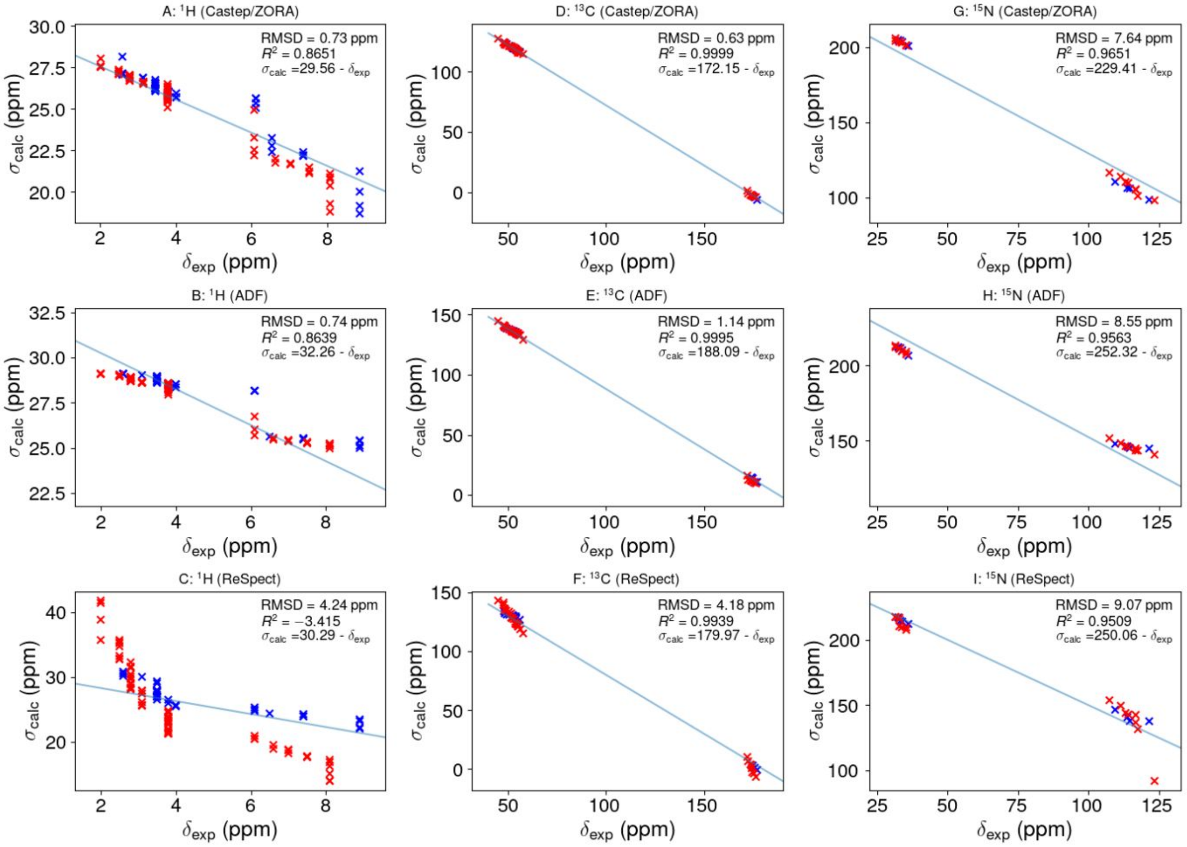


**Figure S8.** Correlations between chemical shielding and chemical shift for Castep/ZORA, ADF, and ReSpect calculations of $^{1}H$, $^{13}C$, and $^{15}N$ NMR parameters. Points represent the experimental data and lines represent the best fits obtained by linear regression on data from both compounds. Color code: red: compound **1**, blue: compound **2**.

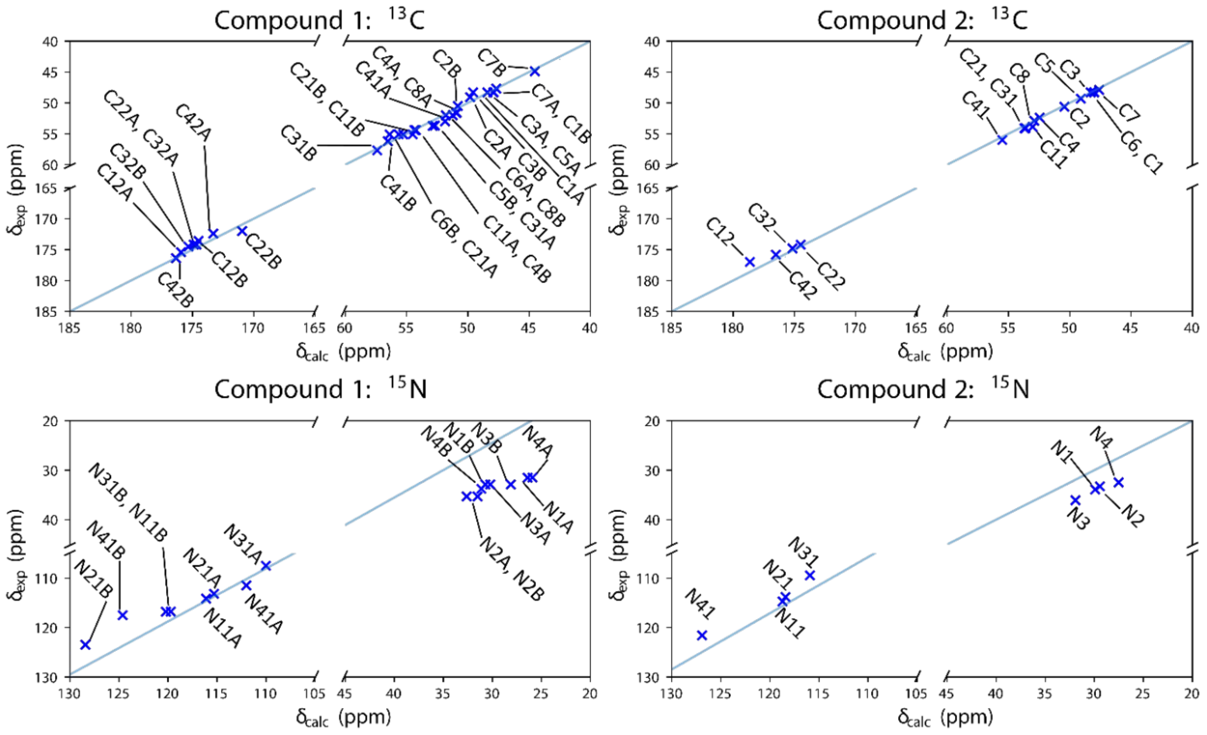


**Figure S9.** Comparison of $^{13}C$ and $^{15}N$ experimental isotropic chemical shifts and isotropic chemical shifts calculated by Castep ($^{13}C$) and Castep with scalar and spin-orbit correction from ReSpect ($^{15}N$) for both compounds. Points represent the experimental data, and the lines show an ideal match between the experimental and calculated values.

**Table S8.** Deconvolution Results of Solid-state $^{13}C$ NMR Spectrum of Compound **1**.

| label(s) | shift (ppm) | height | width (Hz) | L/G | area | integral | number of nuclei |
|---|---|---|---|---|---|---|---|
| | | | Carbonyl region | | | | |
| C12A | 176.4 | 84531 | 101.0 | 0.8 | 5895129 | 1.3 | 1 |
| C42B | 175.4 | 51226 | 126.6 | 0.8 | 4478146 | 1.0 | 1 |
| C32B | 174.5 | 53809 | 70.9 | 0.8 | 2635772 | 0.6 | 1 |
| C22A, C32A | 174.2 | 166353 | 86.0 | 0.8 | 9883661 | 2.2 | 2 |
| C12B | 173.6 | 90491 | 57.0 | 0.8 | 3565596 | 0.8 | 1 |
| C42A | 172.4 | 106398 | 78.5 | 0.8 | 5768966 | 1.3 | 1 |
| C22B | 172.0 | 81704 | 63.3 | 0.8 | 3575092 | 0.8 | 1 |
| | | | Aliphatic region | | | | |
| C31B | 57.7 | 67218 | 66.6 | 0.8 | 3121980 | 0.6 | 1 |
| C41B | 56.2 | 79796 | 86.9 | 0.8 | 4834244 | 0.9 | 1 |
| C6B, C21A, C21B, C11B | 55.1 | 281977 | 101.3 | 0.8 | 19917707 | 3.8 | 4 |
| C11A, C4B | 54.4 | 244063 | 68.5 | 0.8 | 11666108 | 2.2 | 2 |
| C5B, C31A | 53.7 | 172706 | 99.4 | 0.8 | 11970685 | 2.3 | 2 |
| C41A | 53.0 | 126802 | 66.2 | 0.8 | 5856885 | 1.1 | 1 |
| C6A, C8B | 52.0 | 228270 | 70.8 | 0.8 | 11266625 | 2.2 | 2 |
| C4A, C8A | 51.6 | 184249 | 82.2 | 0.8 | 10560638 | 2.0 | 2 |
| C2B | 50.5 | 94031 | 98.2 | 0.8 | 6439119 | 1.2 | 1 |
| C2A | 49.1 | 72376 | 175.2 | 0.8 | 8827963 | 1.7 | 1 |
| C3B, C1A, C3A, C5A | 48.3 | 294544 | 93.7 | 0.8 | 19245503 | 3.7 | 4 |
| C7A, C1B | 47.7 | 124805 | 113.7 | 0.8 | 9891690 | 1.9 | 2 |
| C7B | 44.9 | 73057 | 41.9 | 0.8 | 2136953 | 0.4 | 1 |

**Table S9.** Deconvolution Results of Solid-state $^{15}N$ NMR Spectrum of Compound **1**.

| label(s) | shift (ppm) | height | width (Hz) | L/G | area | integral | number of nuclei |
|---|---|---|---|---|---|---|---|
| | | | Amide region | | | | |
| N21B | 123.4 | 127.12 | 59.74 | 0.8 | 5758 | 0.8 | 1 |
| N41B | 117.5 | 197.64 | 44.62 | 0.8 | 6687 | 1.0 | 1 |
| N31B, N11B | 116.8 | 269.46 | 55.5 | 0.8 | 11339 | 1.6 | 2 |
| N11A | 114.1 | 166.87 | 41.13 | 0.8 | 5204 | 0.7 | 1 |
| N21A | 113.2 | 118.87 | 63.78 | 0.8 | 5749 | 0.8 | 1 |
| N41A | 111.5 | 178.9 | 53.26 | 0.8 | 7571 | 1.1 | 1 |
| N31A | 107.4 | 219.51 | 50.85 | 0.8 | 8463 | 1.2 | 1 |
| | | | Amine region | | | | |
| N2A, N2B | 35.3 | 367.84 | 44.9 | 0.8 | 12524 | 1.8 | 2 |
| N4B | 33.8 | 341.23 | 38.15 | 0.8 | 9871 | 1.4 | 1 |
| N1B, N3A, N3B | 32.9 | 753.2 | 41.74 | 0.8 | 23836 | 3.4 | 3 |
| N1A, N4A | 31.5 | 342.92 | 55.01 | 0.8 | 14305 | 2.1 | 2 |

**Table S10.** Deconvolution Results of Solid-state $^{13}C$ NMR Spectrum of Compound **2**.

| label(s) | shift (ppm) | height | width (Hz) | L/G | area | integral | number of nuclei |
|---|---|---|---|---|---|---|---|
| | | | Carbonyl region | | | | |
| C12 | 177.0 | 180523.1 | 61.67 | 0.8 | 7119051 | 0.8 | 1 |
| C42 | 175.8 | 200826.8 | 54.42 | 0.8 | 6991366 | 0.8 | 1 |
| C32 | 174.8 | 180831.1 | 76.49 | 0.8 | 8846354 | 1.0 | 1 |
| C22 | 174.2 | 187195.8 | 63.25 | 0.8 | 7582869 | 0.9 | 1 |
| | | | Aliphatic region | | | | |
| C41 | 56.0 | 218514.5 | 60.34 | 0.8 | 8447790 | 1.0 | 1 |
| C21, C31 | 54.1 | 448953.5 | 57.16 | 0.8 | 16414912 | 1.9 | 2 |

| | | | | | | | |
|---|---|---|---|---|---|---|---|
| C11 | 53.9 | 371526.6 | 43.58 | 0.8 | 10344860 | 1.2 | 1 |
| C8 | 52.9 | 280949.9 | 64.06 | 0.8 | 11508441 | 1.3 | 1 |
| C4 | 52.4 | 199438.2 | 49.86 | 0.8 | 6386243 | 0.7 | 1 |
| C2 | 50.6 | 256493.4 | 56.26 | 0.8 | 9218125 | 1.1 | 1 |
| C5 | 49.3 | 232953.2 | 58.83 | 0.8 | 8764867 | 1.0 | 1 |
| C3, C6, C1 | 48.3 | 571553.2 | 74.16 | 0.8 | 27112163 | 3.1 | 3 |
| C7 | 47.9 | 350284.1 | 46.25 | 0.8 | 10341395 | 1.2 | 1 |

**Table S11.** Deconvolution Results of Solid-state $^{15}$N NMR Spectrum of Compound **2**.

| label(s) | shift (ppm) | height | width (Hz) | L/G | area | integral | number of nuclei |
|---|---|---|---|---|---|---|---|
| | | | Amide region | | | | |
| N41 | 121.5 | 510.07 | 22.28 | 0.8 | 8615 | 0.9 | 1 |
| N21 | 114.7 | 689.02 | 20.17 | 0.8 | 10540 | 1.1 | 1 |
| N11 | 113.8 | 509.48 | 18.01 | 0.8 | 6956 | 0.8 | 1 |
| N31 | 109.5 | 605.38 | 17.49 | 0.8 | 8026 | 0.9 | 1 |
| | | | Amine region | | | | |
| N1 | 36.1 | 791.09 | 18.16 | 0.8 | 10895 | 1.2 | 1 |
| N3 | 33.9 | 967.36 | 12.98 | 0.8 | 9518 | 1.0 | 1 |
| N2 | 33.3 | 883.31 | 12.88 | 0.8 | 8627 | 0.9 | 1 |
| N4 | 32.4 | 712.15 | 19.02 | 0.8 | 10269 | 1.1 | 1 |

## FITTING PROCEDURE OF $^{199}$Hg NMR SPECTRA

Automated fitting of the static ultra-wideline NMR spectra with multiple sites is often not feasible without further information due to the high sensitivity of the fitting to the initial parameter estimates. Therefore, in the present work, conversion of the shielding components calculated by ADF to NMR shift components according to the published equation ($\sigma_{calc} = -1.3457\ \delta_{exp} + 7853$)[3] was attempted. Nonetheless, the match with the experimental spectra was not satisfactory. In this context, it is noteworthy that ref.[3] itself presents several different calibration equations, depending, among other factors, on the size of the molecular clusters used for the calculations. As in other places in this paper, we preferred to keep the slope of the calibration equation equal to −1 in order not to compensate for errors in the computational scheme by adjustment of the slope. This was necessary because in multiple cases, a severe deviation from the ideal slope was observed. Moreover, the published calibration equations were established only using NMR spectra of inorganic mercury(II) compounds, mainly halogenides. However, in this paper, mercury(II) complexes of organic ligands are studied as well. Therefore, it was necessary to find a new, more appropriate calibration equation.

Despite these problems, the NMR parameters calculated from the ADF results by the above-mentioned calibration equation were still used as the starting point for fitting the experimental spectra. The fitting of the spectra in Topspin provided a good match with the experiment. However, to prevent bias from the chosen starting point, new calibration equations for both ADF and ReSpect were found. In the next step, the $^{199}$Hg NMR spectra were refitted using the midpoint of the NMR parameters from the ADF and ReSpect calculations. The spectra measured on 500 MHz and

700 MHz spectrometers were fit independently. By manual tweaking of the parameters of the final fits, the errors in these parameters were estimated. Finally, the fitting procedure provided a very good match between the fitted spectra and the experimental data and led to refinement of the calibration equations unbiased by a starting point of the spectrum fitting procedure.

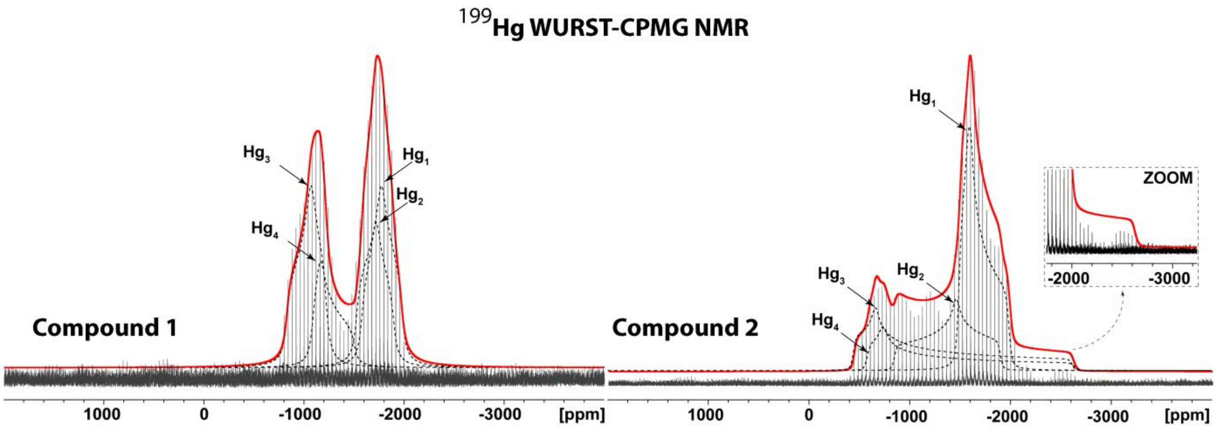


**Figure S10.** Experimental $^{199}$Hg WURST-CPMG ssNMR spectra (black solid line), simulations of the individual mercury sites (dashed lines) and their sum (red solid line) of the of compound **1** (left-hand spectrum) and compound **2** (right-hand spectrum). Both spectra were recorded on a 700 MHz spectrometer.

**Table S12.** Overview of Experimentally Determined Principal Components of Chemical Shifts Tensors and Principal Components of Chemical Shielding Tensors Calculated by ReSpect and ADF for All $^{199}$Hg Sites in Both Compounds. All Values Are in ppm.

| | | exp (500 MHz) | | | exp (700 MHz) | | | ReSpect | | | ADF | | |
|---|---|---|---|---|---|---|---|---|---|---|---|---|---|
| | site | $\delta_{11}$ | $\delta_{22}$ | $\delta_{33}$ | $\delta_{11}$ | $\delta_{22}$ | $\delta_{33}$ | $\sigma_{11}$ | $\sigma_{22}$ | $\sigma_{33}$ | $\sigma_{11}$ | $\sigma_{22}$ | $\sigma_{33}$ |
| compound 1 | Hg1 | −1571 | −1749 | −1972 | −1570 | −1759 | −2000 | 13004 | 13161 | 13476 | 10532 | 10718 | 10949 |
| | Hg2 | −1425 | −1675 | −1832 | −1437 | −1710 | −1833 | 12486 | 12939 | 13275 | 10185 | 10514 | 10786 |
| | Hg3 | −769 | −1052 | −1218 | −759 | −1046 | −1194 | 12211 | 12613 | 12852 | 9709 | 10025 | 10237 |
| | Hg4 | −910 | −1111 | −1425 | −1069 | −1185 | −1533 | 12143 | 12228 | 12761 | 9728 | 9823 | 10277 |
| co mp | Hg1 | −1451 | −1609 | −2037 | −1454 | −1601 | −1988 | 12897 | 12987 | 13402 | 10478 | 10566 | 10955 |

| | | | | | | | | | | | | |
|---|---|---|---|---|---|---|---|---|---|---|---|---|
| Hg2 | −502 | −730 | −2896 | −435 | −636 | −2669 | 11634 | 11894 | 14851 | 9254 | 9363 | 12381 |
| Hg3 | −805 | −1473 | −1958 | −787 | −1457 | −1941 | 11972 | 12732 | 13377 | 9410 | 10210 | 10808 |
| Hg4 | −419 | −642 | −2905 | −546 | −738 | −2675 | 11627 | 11807 | 14777 | 9267 | 9416 | 12378 |

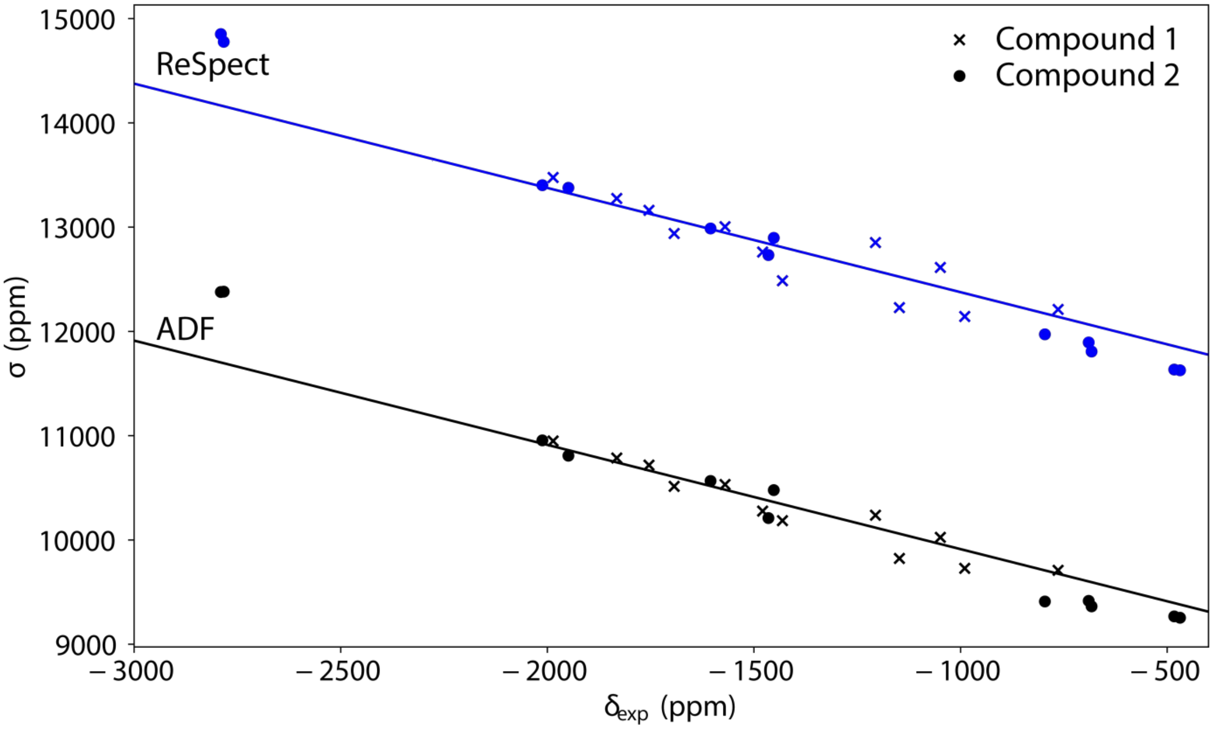


**Figure S11.** Internal calibration of [199]Hg chemical shift based on calculated principal shielding components and experimental principal chemical shift components (averaged data measured on 500 MHz and 700 MHz spectrometer). Calibration curves: $\sigma_{calc}$ (ADF) = 8942 − $\delta_{exp}$; $R^2$ = 0.85, $\sigma_{calc}$ (ReSpect) = 11398 − $\delta_{exp}$; $R^2$ = 0.84.

## LINEAR REGRESSION MODELS OMITTING HEAVY ATOMS FROM THE TRAINING SET

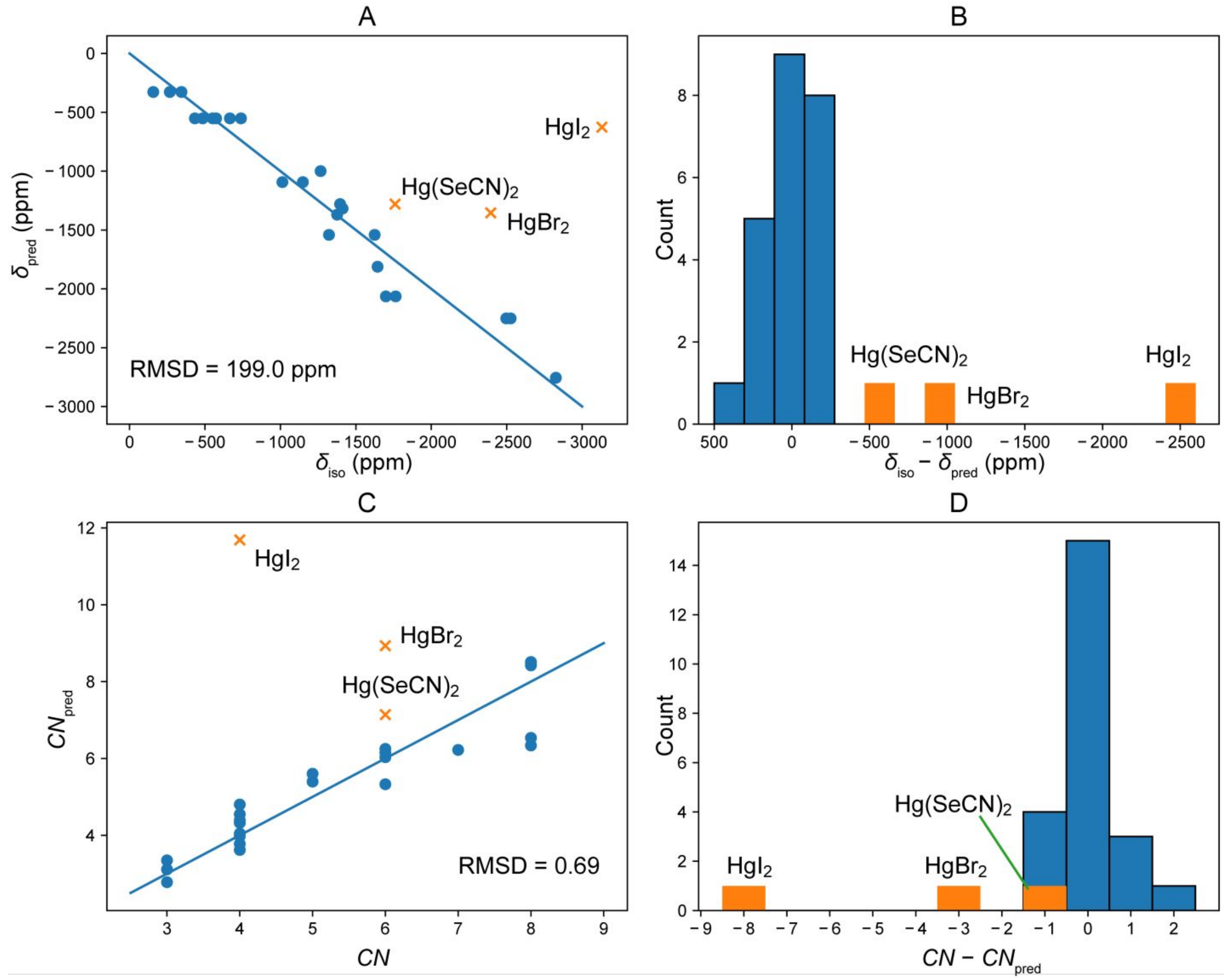


**Figure S12**. **A**: Comparison of experimental isotropic chemical shifts with the shifts predicted by linear regression according to Eq (1) applied on dataset without $HgI_2$, $HgBr_2$, $Hg(SeCN)_2$. Line represents an exact match. **B**: histogram of prediction errors. Regression equation $\delta_{pred} = -224.11\ CN - 934.204\ \bar{\chi} + 2755.17$ was found by linear regression with adjusted $R^2 = 0.94$. **C**: Comparison of the X-ray coordination number with the coordination number predicted by linear regression according to Eq. (2) applied on dataset without $HgI_2$, $HgBr_2$, $Hg(SeCN)_2$. **D**: histogram of prediction errors. Regression equation $CN_{pred} = -0.0030\ \delta_{iso} - 1.7145\ \bar{\chi} + 6.7243$ was found by

linear regression with adjusted $R^2$ = 0.86. RMSDs were calculated by leave-one-out cross validation on datasets excluding $HgI_2$, $HgBr_2$ and $Hg(SeCN)_2$.

Finally, an attempt was made to find a correlation between the span and the variability of the Hg–X coordination bond lengths expressed as population standard deviation. However, a clear trend was only observed for compounds with the more regular coordination polyhedra with coordination numbers 4 or 8. Furthermore, no general equation describing the relationship could be obtained since the slope of the function of span vs. standard deviation of the bond lengths appears to be dependent on the coordination number, see **Figure S13**.

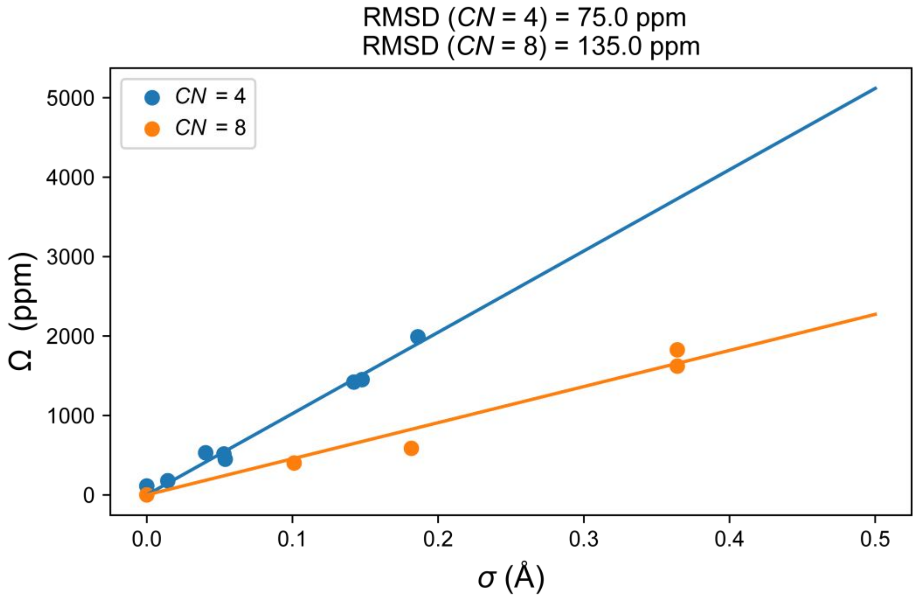


**Figure S13.** Dependance of span (Ω) on standard deviation of the Hg–X bond lengths (σ). Only compounds with coordination number 4 and 8 exhibited clear linear relationship. Points represent the experimental values and lines represent the best fits obtained by linear regression. Regression equations: $CN = 4$: $\Omega = 10200 \cdot \sigma$; $CN = 8$: $\Omega = 4500 \cdot \sigma$. The offset of linear function was

originally found to be insignificantly different from zero (at 50% level of importance), therefore, it was ommited in the final fitting.

## APPLICATION OF THE OBTAINED MODELS FOR SOLUTION STATE SYSTEMS

The applicability of the obtained models would be greatly increased by including solution state systems in the training set. Though this extension is outside the scope of this paper, in this section we briefly test the prediction accuracy on solution systems described in literature.[4] Specifically, we test two approaches here. First, we assume that the DFT-calculated structure is a realistic representation of the situation in the solution. Thus, $[Hg(do4s)]^{2+}$ and $[Hg(do3s)]^{2+}$ contain a hexacoordinated Hg(II) ion (DFT-calculated Hg–S distances for two and one pendant arms are non-bonding in each structure, respectively). However, strong coordination of nitrate to $[Hg(cyclen)]^{2+}$ is unlikely in solution, thus Hg(II) ion in this structure will be considered as tetracoordinated. Results are summarized in **Table S13**, showing prediction errors slightly higher than for the cross-validation RMSDs for the solid-state training set.

**Table S13**. Test of the Suitability of the Models for Solution-State System.

| | $\delta_{iso}$ (ppm)[a] | $CN$[b] | $\bar{\chi}$ | $\delta_{pred}$ (ppm)[c] | $\delta_{pred2}$ (ppm)[d] | $CN_{pred}$[c] | $CN_{pred2}$[d] |
|---|---|---|---|---|---|---|---|
| $[Hg(do4s)]^{2+}$ | −1258 | 6 | 2.89 | −1387 | −1286 | 4.73 | 5.55 |
| $[Hg(do3s)]^{2+}$ | −1160 | 6 | 2.89 | −1387 | −1286 | 4.53 | 5.26 |
| [Hg(do2a2s)] | −1413 | 8 | 3.03 | −2022 | −1864 | 5.02 | 5.78 |
| $[Hg(dota)]^{2-}$ | −1828 | 8 | 3.24 | −2191 | −2065 | 5.83 | 6.65 |
| $[Hg(cyclen)]^{2+}$ | −1117 | 4 | 3.04 | −981 | −981 | 4.43 | 4.86 |
| RMSD | | | | 343 | 243 | 1.87 | 1.29 |

[a]Experimental chemical shift.[4] [b]Deduced from DFT calculations.[4] [c]Model containing $HgBr_2$ and $Hg(SeCN)_2$ in the training set. [d]Model without $HgBr_2$ and $Hg(SeCN)_2$ in the training set.

However, the “true“ solution structure is often a dynamic structure or an equilibrium between several conformers, which are not necessarily the lowest energy ones. We can suppose that Hg(II) ion in $[Hg(dota)]^{2-}$ complex is not purely octacoordinated due to the dynamics (as was evidenced by VT NMR) and the same is likely the case for [Hg(do2a2s)] complex (evidenced by significantly longer Hg–O distances than in $[Hg(dota)]^{2-}$).[4] These dynamics lower the effective coordination number of Hg(II) ions in these complexes. For this reason, we also try to account for the dynamics by lowering the *CN* in this analysis, see **Table S14**. The RMSD for both the predicted chemical shift and the predicted coordination number significantly improved. Although the match between the experimental and predicted parameters is not ideal for the set of solution data, this example still illustrates the potential. We must highlight the fact that the model training was not done on solution state data and thus the error can also be of systematic nature, which could be mitigated by re-training the models on solution state data.

**Table S14.** Test of the Suitability of the Models for Solution-State System, Accounting for the System Dynamics by Lowering the Effective Coordination Number.

| | $\delta_{iso}$ (ppm)[a] | $CN$[b] | $\bar{\chi}$ | $\delta_{pred}$ (ppm)[c] | $\delta_{pred2}$ (ppm)[d] | $CN_{pred}$[c] | $CN_{pred2}$[d] |
|---|---|---|---|---|---|---|---|
| $[Hg(do4s)]^{2+}$ | −1258 | 6 | 2.89 | −1387 | −1286 | 4.73 | 5.55 |
| $[Hg(do3s)]^{2+}$ | −1160 | 6 | 2.89 | −1387 | −1286 | 4.53 | 5.26 |
| [Hg(do2a2s)] | −1413 | 7 | 2.917 | −1712 | −1584 | 5.03 | 5.88 |
| $[Hg(dota)]^{2-}$ | −1828 | 7 | 3.21 | −1905 | −1814 | 5.83 | 6.70 |
| $[Hg(cyclen)]^{2+}$ | −1117 | 4 | 3.04 | −981 | −981 | 4.43 | 4.86 |
| RMSD | | | | 191 | 114 | 1.36 | 0.75 |

[a]Experimental chemical shift.[4] [b]Deduced from DFT calculations.[4] [c]Model containing $HgBr_2$ and $Hg(SeCN)_2$ in the training set. [d]Model without $HgBr_2$ and $Hg(SeCN)_2$ in the training set.

## MOLECULAR ORBITAL ANALYSIS DIAGRAM

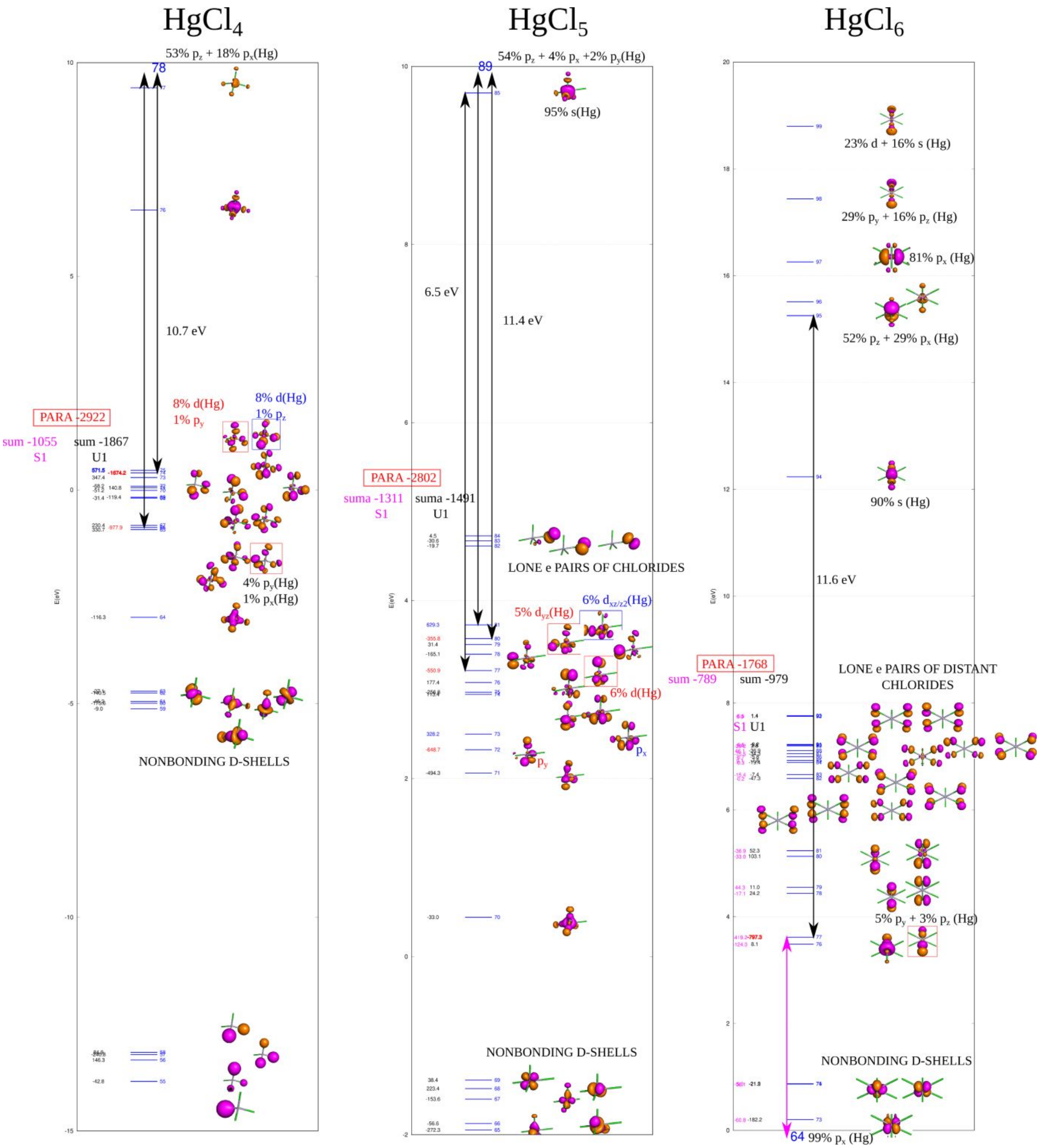


**Figure S14.** Molecular orbital analysis diagram for a series of clusters $(HgCl_n)^{2-n}$ ($n$ = 4, 5, 6) calculated by ADF.

The analysis of paramagnetic contribution to isotropic NMR shielding of $^{199}$Hg was performed on scalar-relativistic canonical MOs in the range HOMO-(HOMO-20) using ADF2024. The blue

lines correspond to electronic energy of the individual MO levels (in eV). The occupied MOs are characterized with U1 values referring to the sum of all magnetic couplings from given MO (black numbers). For $(HgCl_6)^{4-}$ also S1 contributions are shown in magenta. Total paramagnetic contributions (in red frame) from studied energetic region follow qualitatively the trend observed for total NMR shielding increasing from $n = 4$ to $n = 6$. The examples of the largest NMR couplings with specified vacant MOs are depicted by arrows. Here, it is worth noting a dominant role of orthogonal *p*-type Hg atomic orbitals (see framed figures of MO wavefunction with indicated population of *p*-orbitals). Both the number and magnitude of the key deshielding transitions (red numbers next to energy levels) drops with increasing number of frontier MOs constituted by different combinations of Cl lone pairs. Lower efficency of occ-vac magnetic couplings in the case of $(HgCl_6)^{4-}$ results in a larger shielding (smaller paramagnetic deshielding) of the Hg nucleus.

REFERENCES

1. ref. 34 in the main text
2. ref. 17 in the main text
3. ref. 33 in the main text
4. ref. 25 in the main text
